# Enhancing HACCP Decisions: A Comparative Risk Assessment for Table Olive Processing

**DOI:** 10.3390/foods15122153

**Published:** 2026-06-14

**Authors:** Cristina Campanero Pintado, Kharla Andreina Segovia Bravo, Antonio Benítez Cabello, Francisco Noé Arroyo-López, Efrén Pérez-Santín

**Affiliations:** 1 Escuela Superior de Ingeniería y Tecnología (ESIT), Universidad Internacional de La Rioja (UNIR), Avenida de la Paz, 137, 26006 Logroño, Spain; 2 Instituto de la Grasa, Consejo Superior de Investigaciones Científicas, Campus Universitario Pablo de Olavide, Edificio 46, Carretera de Utrera, km 1, 41013 Seville, Spain

**Keywords:** table olives, HACCP, risk assessment, food safety, critical control points

## Abstract

Table olive processing comprises multiple stages in which physical, chemical, and biological hazards may occur. Although risk assessment is a core element of Hazard Analysis and Critical Control Points (HACCP) systems, the selection of assessment tools remains insufficiently standardized. This study compared a 4 × 4 risk matrix and Failure Mode and Effects Analysis (FMEA) for hazard evaluation in Spanish-style and Californian-style table olive processing. Hazards were assessed across 41 processing stages for Spanish-style olives and selected key stages for Californian-style olives using probability × severity in the 4 × 4 matrix and severity × occurrence × detection in FMEA. Significant hazards were further evaluated using the Codex Alimentarius decision tree to identify critical control points (CCPs) and strengthened prerequisite programs (PRPs). Both tools identified similar significant hazards, including biological hazards associated with fermentation, brine management, storage, container sealing, and heat treatment, as well as physical hazards from foreign bodies and chemical hazards related to heavy metals, pesticide residues, mycotoxins, and food-contact material migration. FMEA provided greater analytical detail through the detection parameter, whereas the 4 × 4 matrix was simpler and more practical for complex flow diagrams. Overall, both tools were suitable for HACCP-based risk assessment in table olive processing.

## 1. Introduction

Table olives constitute a key component of the Mediterranean basin’s culinary and cultural traditions and are currently the most widely produced and consumed fermented vegetable product in the region. According to the International Olive Council [1], global production reaches approximately 3.1 million tons per year, making table olives the leading fermented vegetable product in the Mediterranean countries.

Among the most widely consumed presentations are Spanish-style green olives (also known as Sevillian-style) and Californian-style black olives, which together represent approximately 85–90% worldwide production. Spanish-style olives are harvested, either manually or mechanically, at the green to yellow–green ripening stage. The fruits are then immersed in a diluted sodium hydroxide (NaOH) lye solution to hydrolyze oleuropein, the main bitter compound. After lye treatment, olives are washed with water to remove excess alkali and transferred into sodium chloride (NaCl) brine, where fermentation takes place, primarily driven by lactic acid bacteria (LAB), although yeasts also play a role in the process [2]. On the contrary, the production of Californian-style black olives, although varying slightly between manufacturers, involves several key stages to achieve the desired sensory characteristics. As with Spanish-style olives, fruits are harvested at the green to yellow–green stage and subsequently stored for several months in acidified solutions (with or without air flow) to preserve firmness and prevent spoilage. Then, during the darkening phase, polyphenols naturally present in the olive are oxidized and polymerized—a critical step responsible for the characteristic black color. Finally, the olives are packed and subjected to thermal sterilization to ensure microbiological safety and extend shelf life [3].

Alerts related to the quality and safety of table olives are relatively rare in the European and U.S markets. However, incidents still occur, and physical, biological and chemical contaminations are possible. Between 2020 and 2025, the European Rapid Alert System for Food and Feed [4] recorded 26 notifications related to table olives. The most frequently reported non-compliances concerned pesticide residues, heavy metals, microbiological agents, and food additives [5]. In the case of the USA, no notifications related to table olives were reported between 2020 and 2025; however, an urgent public health warning regarding the potential contamination of imported table olives with Clostridium botulinum, was reported by the FDA in 2007 [6]. Formerly, Valero et al. [7] reviewed the microbial hazards and their implications in the production of table olives, paying special attention to biological risk management.

All these factors highlight the need to implement effective risk management systems within companies operating in the olive sector. Food risk management is a key component in the strategic planning of any olive business committed to ensuring the safety of its products and protecting public health. In a context marked by increasing regulatory complexity and growing societal demands for transparency and accountability, the design and implementation of effective systems for the identification, assessment, and control of food-related hazards is not only a legal requirement but also a critical factor for market differentiation and competitiveness.

The Hazard Analysis and Critical Control Points (HACCP) framework is widely acknowledged as a foundational element in global food safety systems. Initially developed in the 1960s by NASA in collaboration with Pillsbury to maintain food safety standards during spaceflight operations, HACCP has since gained international endorsement from organizations such as the Codex Alimentarius Commission, the World Health Organization (WHO), and the Food and Agriculture Organization (FAO). Today, its principles are extensively implemented throughout the food industry to proactively identify, assess, and manage potential hazards that could affect product safety [8].

Beyond being a best practice, HACCP is also a regulatory requirement in many regions. For instance, within the European Union, food businesses are legally obligated to apply HACCP-based procedures under Regulation (EC) No 852/2004 [9], which governs hygiene standards across all stages of food production and distribution. Moreover, voluntary certification schemes such as ISO 22000, FSSC 22000, IFS Food, and BRCGS Food also mandate the use of HACCP principles. These standards require companies to systematically identify key hazards and establish critical control points (CCPs) to ensure the safety and integrity of their food products [8].

In the realm of food production, a structured risk analysis process is essential to ensure product safety. This process typically involves three key steps: 1. identifying hazards and reviewing existing control measures, 2. assessing risks by evaluating the likelihood and severity of each hazard, and 3. managing risks through the implementation of appropriate controls, such as CCPs and stricter prerequisite programs (PRPs). This methodology forms the backbone of any effective HACCP system [10].

The first step, hazard identification and review of current controls, is generally well-supported by scientific literature and technical guidelines tailored to specific food categories. Likewise, the third step, defining control measures, is relatively straightforward once a significant hazard has been identified, as industry standards and best practices offer clear mitigation strategies for a wide range of food safety risks. However, the second step, risk assessment, often presents considerable challenges. A major issue is the lack of standardized guidance on how to select and apply risk matrices. The absence of explicit guidance in both legislation and voluntary standards regarding the selection of risk assessment matrices for food-related processes introduces a degree of subjectivity into the evaluation. Consequently, the final risk classification may be influenced by the assessor’s background, experience, and decision-making approach.

The subjective nature of risk matrix selection introduces potential bias into decision-making processes, which can compromise both the objectivity and reproducibility of HACCP systems. In many cases, risk assessments are approached as mere compliance exercises, rather than being utilized as practical tools for enhancing food safety. Their true effectiveness depends largely on how well they are integrated into operational practices and decision-making frameworks [11].

In the absence of a well-established methodology for selecting risk matrices, decision-making may rely heavily on subjective perceptions and professional judgment rather than on objective, evidence-based criteria. This can lead to inconsistencies in hazard evaluation and may result in underestimating or overlooking critical food safety risks [12]. Given this challenge, there is an urgent need for scientific research and regulatory guidance to fill the gap by establishing standardized criteria for selecting appropriate risk matrices and defining robust evaluation methods tailored to different food categories and processing contexts. In light of these limitations, this study seeks to provide guidance for the table olive industry by comparing the performance of several risk matrices when applied to green and black table olive production processes. The goal is to improve and update the existing Quality Management Guide for the Table Olive Industry (T.OT/Doc. No. 14) [13] through the identification of which risk matrix provides the most effective framework for assessing risks in this application, thereby improving the consistency, transparency, and scientific rigor of decision-making within HACCP planning in table olive processing.

The novelty of the present study lies in three key aspects. First, it provides a direct comparative application of two structurally different risk assessment approaches (4 × 4 qualitative risk matrix and the semi-quantitative FMEA method) under identical processing conditions. Second, the study evaluates these methodologies under real processing conditions for both Spanish-style and Californian-style table olives, contributing practical evidence to a sector with limited comparative research. Third, it integrates the outputs of both risk assessment methods with the Codex Alimentarius decision tree to support the systematic identification of CCPs and stricter PRPs. This combined approach contributes to improving consistency, transparency, and scientific robustness of risk assessment within HACCP systems, particularly in complex food processing chains.

From this perspective, the primary contribution of this study is methodological, as it evaluates and compares two widely used risk assessment tools within a HACCP framework. However, this methodological contribution is supported by its application to a specific industrial sector (table olive processing) providing practical validation under real operational conditions.

## 2. Materials and Methods

### 2.1. Case Study Description

The case study was conducted in a medium-sized agro-industrial enterprise based in Seville (Spain), dedicated to the processing and international commercialization of the two predominant table olive products: green Spanish-style olives and black Californian-style olives. The industrial plant occupies an area of approximately 8500 m^2^ and is capable of processing up to 15,000 tons of olives annually. The company serves both domestic and international markets, exporting a substantial proportion of its production to various European countries. Descriptions of raw and auxiliary materials, process flowchart, and descriptions of the process’s stages for both products (Spanish-style and Californian-style table olives) are available in the Supplementary Data (see Sections S1–S3, Figures S1 and S2).

This case study is used as a representative industrial framework to apply and compare the selected risk assessment methodologies, rather than as the primary focus of the research itself.

### 2.2. Control Measures Implemented

The control measures currently in place within the facility are summarized below: Supplier qualification and approval program designed to ensure the safety of all incoming raw materials and ingredients. Olive suppliers are subjected to an annual audit and present a crop certificate for each batch about pesticide, heavy metal and mycotoxin analysis, as well as microbiological analysis for the detection of the main pathogens linked to crops.

Reception control plan to guarantee that all inputs received meet the specifications agreed with the supplier in terms of safety and quality.

Water control plan including chemical and microbiological analysis to ensure its quality and safety.

Cleaning and disinfection plan including only authorized cleaning and disinfecting agents.

Preventive maintenance plan.

Pest control plan.

Training plan.

Good manufacturing practices.

### 2.3. Risk Assessment Methodology

The hazards identified in the previous stage were evaluated using two distinct risk assessment approaches. The first consisted of a 4 × 4 risk matrix that combines probability and severity criteria, adapted from the methodology proposed by Surareungchai et al. [14]. The second approach was based on the risk evaluation procedure employed in Failure Mode and Effects Analysis (FMEA), following the framework described by Cartín-Rojas et al. [15].

A panel of three evaluators with expertise in food safety risk analysis, HACCP systems and table olive processing conducted the risk assessment. The evaluation process was collaborative. The identification of hazards and the assignment of scores for severity, likelihood of occurrence and detection were discussed and agreed upon based on scientific literature [16,17,18,19,20,21,22,23,24,25,26,27,28,29,30,31,32], applicable legislation [9,33,34,35,36,37,38], sector-specific guidelines [1,5] and historical data from table olive processing operations, as well as other references cited in this article. This approach ensured consistency in the scoring process and minimized subjectivity in the evaluation. In cases of initial discrepancy, agreement was reached through discussion and consensus among the evaluators, supported by these sources, to ensure the criteria were aligned.

It should be noted that the risk assessment performed in this study follows a semi-quantitative approach, widely used in HACCP systems. The parameters of severity, likelihood of occurrence, and detection were not derived from quantitative or probabilistic datasets but instead were assigned using predefined categorical scales for each method. Therefore, the probability values represent qualitative likelihood categories rather than statistically measured frequencies.

#### 2.3.1. 4 × 4 Risk Matrix

A 4 × 4 Risk Matrix (Supplementary Data, Table S1), adapted from the model proposed by Surareungchai et al. [14], was applied for hazard evaluation. The assessment was based on two key criteria: the severity of the potential consequences and the likelihood of hazard occurrence.

Hazard significance was subsequently determined to identify process-stage hazards that could adversely affect product safety or pose a risk to consumer health. For this purpose, hazards classified as either medium- or high-risk were considered significant and therefore required further attention within the risk management process.

#### 2.3.2. FMEA Methodology

The risk assessment approach based on Failure Mode and Effects Analysis (FMEA), as described by Cartín-Rojas et al. [15], was also applied. Under this methodology, each identified hazard was evaluated according to three criteria: severity, probability of occurrence, and detectability (Supplementary Data, Table S2). As in the 4 × 4 matrix approach, severity and occurrence scores were assigned using information gathered from scientific literature [16,17,18,19,20,21,22,23,24,25,26,27,28,29,30,31,32], legislation [9,33,34,35,36,37,38] and sector-specific guidance documents [1,5]. The detectability score was determined by considering the effectiveness of the control measures already implemented by the company for each identified hazard.

Subsequently, the Criticality Index (CI) was calculated using the following formula:

CI = S × O × D

where

S = Severity of risk

O = Likelihood of occurrence

D = Likelihood of detection

After calculating the Criticality Index (CI) for each identified hazard, the classification criteria proposed by Cartín-Rojas et al. [15] were used to distinguish significant hazards from those considered non-significant. Hazards with a CI value of 25 or higher, as well as those assigned a high severity rating (categories 4 or 5), were classified as significant and therefore required particular attention within the risk assessment process.

#### 2.3.3. CCP and Stricter PRP Determination

Once the risk evaluation had been carried out with both approaches, the hazards identified as significant were subjected to the Codex Alimentarius decision-tree procedure [39]. The objective was to establish the most appropriate control strategy, distinguishing between hazards requiring management through CCPs and those adequately controlled by strengthened PRPs.

## 3. Results and Discussion

### 3.1. Spanish-Style Processing

#### 3.1.1. Hazard Identification

Identifying hazards associated with products and processes remains a significant challenge for the food industry, as it requires an extensive and often fragmented literature review, as well as expert knowledge to interpret and prioritize relevant risks [40]. This step can therefore become a bottleneck in HACCP plan development, particularly in complex processing systems such as table olive production.

To support the olive industry, a structured and updated overview of the main hazards associated with its products and processes is provided below, facilitating a more systematic approach to hazard identification.

In the flowchart of the Spanish-style for pitted Manzanilla olives packed in pasteurized glass are identified 41 stages in total (Supplementary Data, Figures S1 and S2). This study includes a comprehensive and detailed analysis of all food safety hazards at each of 41 stages (Supplementary Data, Table S3). Nevertheless, Table 1 provides a representative subset of the results derived from the phases identified as most relevant in terms of food safety hazard assessment.

Regarding physical hazards, branches and leaves and metals from machinery are common foreign bodies found in the food supply chain, including the table olive process [41,42,43]. To mitigate these risks, it is essential for facilities to implement preventive maintenance as a prerequisite program. However, although this prerequisite program is imperative, this process also incorporates metal and X-ray detectors as effective mitigation measures against metal and non-metal foreign bodies, as previously outlined by Onyeaka et al. [44]. Finally, the breakage of glass containers after olive packaging is another hazard that must be considered. To mitigate this risk, it is essential that the organization implements good manufacturing practices to reduce the possibility of passing glass splinters into the product.

Regarding chemical hazards, heavy metals, and pesticide presence, risks were determined in the reception stage. In this sense, Taghizadeh et al. [45] reported pesticides and heavy metals as potential threats associated with olives. Several authors [45,46,47,48,49] associated these compounds with important health concerns. To reduce the human risk, the European Union has regulated the use of pesticides [5] and the maximum level allowed of cadmium in table olives [36].

Likewise, Chen et al. [50] also described the presence of heavy metals in water due to environmental contamination. To reduce this risk in washing, transport and flotation stages, the water control plan must be aligned with European and Spanish regulations [38,51].

Finally, mycotoxin presence risk in the reception stage was detected. These secondary metabolites produced by toxigenic fungi [52] are responsible of mutagenic and carcinogenic processes [40]. The supplier approval plan and the reception control reduce these hazards. Another issue that must be considered in this process is the chemical migration due to packaging material. Although glass is considered a more inert material than others like plastic ones [53,54], it can also release inorganic contaminants into food, such as lead [55]. As the chemical migration is associated with potential health risks [56], the European Union has published several regulations about it [33,34]. However, it is worth noting that glass does not have any specific regulation in the European Union, as if it happens with plastic materials [37]. To reduce this potential contamination, the facility has implemented a supplier approval plan and a reception control plan.

On the other hand, the use of cleaning agents in the food industry is essential to guarantee food safety. According to European legislation, particularly the Hygiene Package [9] and the Biocidal Products Regulation [57], only authorized cleaning and disinfecting agents can be used in food processing environments. Improper cleaning and disinfection practices can lead to contamination of food, potentially harming consumer health. Therefore, it is important to consider this hazard during the process to ensure effective control and minimize the risk of cleaning and disinfection agent residues in food [58].

Olives undergo an alkaline treatment with lye to reduce their bitterness [59]. Although lye is a highly toxic chemical, due to the characteristics of the treatment, it is not considered a food safety hazard. In fact, two reliable entities in this field, such as ASEMESA [60] and International Olive Council [13], do not consider the lye treatment as a stage with food safety impact if the NaOH applied is food grade. To guarantee this, the supplier control plan is imperative. Furthermore, much of the residual NaOH that may remain in the fruit is subsequently neutralized during the subsequent lactic fermentation process.

Concerning biological hazards, risks were determined in several stages of the process. The presence of foodborne pathogens in table olives is not usual. However, several studies have documented the presence of *Listeria monocytogenes* [4,17,20] and evaluated its survival during various stages of processing and storage [22,23,26,28,30,31,32]. Other food-borne pathogens also detected during table olive processing include *Staphylococcus aureus* [16,29], as well as members of the *Enterobacteriaceae* family, such as *Yersinia enterocolitica* and *Escherichia coli* [16,21,25]. Despite their detection, there are no documented outbreaks of illness directly attributed to these microorganisms in Spanish-style green table olive processing.

Nevertheless, Nguyen-The [61] named *Salmonella*, *E. coli* O157, *L. monocytogenes*, *Bacillus cereus* and *Clostridium* spp. as the most common bacteria present on raw fruits and vegetables. It is worth noting that all these microorganisms are responsible for important health concerns as previously described by Singh et al. [40]. For this threat, olive washing to remove organic materials and pathogen microorganisms on their surface must be implemented. However, the quality of water must also be controlled to guarantee the microbial safety of these stages. Singh et al. [40] reported that water is a potential source of microbial contamination of food. As described above, the water plan control complies with Royal Decree 3/2023 [38]; therefore, these threats are under control.

On the other hand, the potential hazards associated with the incorrect use of cover brine must also be analyzed. Brine is a NaCl solution (typically 8–12% in table olives) essential to guarantee a correct fermentation process with the absence of spoilage and food-borne pathogens. LABs are also involved in the later fermentation process, an imperative stage for obtaining a safe and quality final product. Therefore, the correct dosage of brine impact on the food safety of the table olives as previously described by Mantzouridou et al. [62]. In addition to the use of brine in this pre-fermentation stage, brine is also added to olives as a preservative of the final product before the containers are hermetically sealed [63]. For this reason, a correct NaCl concentration (4–5%), pH (<4.3), and acidity (0.3–0.5) of the cover brine solution must be guaranteed.

Along with the addition of brine, the fermentation process is key to obtaining table olives. Perpetuini et al. [64] define olive fermentation as a complex process that involves a diverse range of microorganisms, primarily LAB (such as *Lactiplantibacillus plantarum* and *Lactiplantibacillus pentosus*) and various yeasts (including *Saccharomyces cerevisiae*, *Wickerhamomyces anomalus*, *Pichia manshurica*, and *Candida boidinii*, among others). Their enzymatic functions contribute to the organoleptic and safety properties of the final product. In this context, LAB are essential for lowering the pH of the brine, creating an environment that inhibits the growth of spoilage and pathogen microorganisms. Therefore, during the fermentation stage, storage in fermentation vessels, fruit conditioning after storage, fruit reception and discharge, and control of pH and NaCl concentration must be maintained to guarantee the product’s safety.

Regarding microbial hazards in the final product, container sealing [40,65] and pasteurization [28,66] play an imperative role in this product. To ensure this target, control of hermetic sealing, temperature, time, and pressure values of pasteurization must be strictly controlled in this process.

Finally, machinery used in this process can also present microbiological contamination. In this sense, the cleaning and disinfection practices are designed to remove dirt and microbes, including biofilms, so this prerequisite program is essential to guarantee the production of safe foods [58,67].

#### 3.1.2. Risk Matrix Comparison

Organizations usually have difficulties carrying out risk assessments because of the wide variety of risk assessment tools available. To assist facilities dedicated to the production of table olives in this decision, this work presents a comparison between two risk assessment methods prior to the identification of CCPs: a 4 × 4 risk matrix model based on probability and severity adapted from Surareungchai et al. [14] and a risk assessment used in the FMEA method as described by Cartín-Rojas et al. [15]. Both methods have been recommended to approach the risk assessment in the context of a HACCP system as previously described by FAO and WHO [39], Szczyrba et al. [68] and Wu & Hsiao [69].

These methods were selected for this study due to several differences between them, which may be interesting to consider in a production process such as the production of table olives. First, the FMEA method includes three variables, the probability of the hazard occurring, its severity, and the probability of detection by the system [15,68], while the 4 × 4 matrix model only includes the probability of occurrence of the hazard and its severity [14]. In addition to this main difference, although methods consider both the probability of occurrence of the hazard and its severity, both matrices describe the risk values of parameters differently [14,15]. This study includes a comprehensive and detailed analysis of all food safety hazards risks at each of 41 stages after applying both risk assessment tools (Supplementary Data, Table S4). Nevertheless, Table 2 provides a representative subset of the results derived from the phases identified as the most relevant in terms of food safety hazard risk assessment.

As shown in Table 2, the 4 × 4 risk matrix model considers significant hazards as both those identified as medium and those identified as major. In this sense, three hazards were identified as medium risk in this process: (i) possible chemical migration from glass packaging; (ii) incorrect sodium hydroxide (NaOH) and salt (NaCl) reception, and (iii) presence of heavy metals in the washing (I), washing (II), transport channels and flotation stages.

Regarding chemical migration from glass, this must be considered a significant hazard. Khokhar & Pawar [70] reported the chemical migration from food packaging materials as a significant concern for food safety and public health. In the specific case of glass, due to the risk of migration of a heavy metal such as cadmium, together with the possibility of this danger occurring if it is not adequately controlled, its categorization as a significant hazard is justified [49,70]. On the other hand, the presence of heavy metals in water is another significant risk. As it is a chemical hazard, the control in the productive process is imperative because consumers have no opportunity to reduce them [71]. Moreover, as previously described by Singh et al. [40], heavy metals are toxic in very low amounts; hence, they must be treated as an important health hazard. In addition, water is a frequent source of these compounds [45].

Respecting hazards categorized as major, a total of three general hazards were identified: (i) foreign bodies at all stages of the process; (ii) heavy metals, pesticides and mycotoxin in olive receiving, and (iii) microorganisms in olive receiving, washing (I), washing (II), fermentation, storage in fermentation vessels, fruit conditioning after storage, fruit reception and discharge, transport channels, flotation, brine addition, container sealing, and pasteurization.

Regarding foreign bodies, these hazards must be categorized as significant because of the characteristics of the food under study and its production process. As previously described by Onyeaka et al. [44], physical hazards can occur from the supply stage to processing, packaging, and distribution. In this context, olives are a small ready-to-eat food that may contain small foreign bodies of a metallic nature (mainly from equipment) and non-metallic nature (such as small stones, leaves, or remains of the olive pits themselves that have not been properly removed). These characteristics, combined with the fact that the food is exposed to the environment throughout its processing until packaging, increase the risk of product contamination. Furthermore, once the olives are packaged, there is a risk of breakage of the container due to the fragile material, such as glass. Therefore, monitoring for foreign bodies is essential [44]. In this sense, four control measures must be implemented and monitored exhaustively to reduce the probability of occurrence: a preventive maintenance plan, good handling practices, metal detector, and X-ray detector.

Regarding chemical hazards associated with olive receiving, they should also be considered significant hazards because their occurrence is possible in olives and the impact on consumer health is high in the long term, as previously described by Taghizadeh et al. [45] about heavy metals and pesticides and Cammerata et al. [52] about mycotoxins.

Respecting microbiological hazards, microorganisms associated with raw material and water used at different stages, such as washing (I), washing (II), transport channels, and flotation, must be considered as significant risks. Nguyen-The [61] and Singh et al. [40] reported that the frequent microbial contamination of raw vegetables and water linked to microorganisms are responsible for important health concerns. Therefore, both the likelihood of occurrence and the severity of a potential contamination in this product justify their categorization as significant hazards.

On the other hand, although olives are considered a stable food due to the fermentation process [64], there are some studies that indicate the possible survival of pathogens despite their low pH, low aw, and high salt concentration of the final product [27]. Therefore, a heat treatment must be applied to reduce the risk. In this sense, pasteurization is a good choice to guarantee the safety of olives [28,66], but the parameters of this control measure must be exhaustively monitored.

After applying the 4 × 4 risk matrix model, the risk assessment was conducted by the FMEA method proposed by Cartín-Rojas et al. [15]. As described by this author, hazards with a CI ≥ 25 were considered significant hazards, as well as hazards with high severity values (categorization value of 4–5). Only one hazard obtained a CI value of 75 (CI ≥ 25): glass splinter associated with labeling, palletizing, storage and shipping stages. Therefore, this hazard was categorized as significant. This value is so high mainly due to the severity of the risk on consumer health as described above and the lack of hazard detection measures in the system since the X-ray detector is available in earlier stages.

The rest of the significant hazards identified obtained a CI < 25, but a severity value of 5, a value considered too high to allow its possible occurrence [15]: (i) foreign bodies throughout the entire production process; (ii) heavy metals, pesticides and mycotoxins in olive receiving; (iii) chemical migration in packaging reception; (iv) incorrect chemical in reception of sodium hydroxide (NaOH) and salt (NaCl); (v) heavy metals in washing (I), washing (II), transport channels and flotation; and vi) microorganisms in olive receiving, washing (I), washing (II), fermentation, storage in fermentation vessels, fruit conditioning after storage, fruit reception and discharge, transport channels, flotation, brine addition, container sealing and pasteurization. As described above, all these hazards are associated with an important health injury; hence, they should be categorized as significant hazards.

This decision was based on the main objective of a food safety system: the protection of consumer health. It is important to consider that zero risk is essentially unattainable, and therefore residual risk must always be considered. In this context, hazards associated with very high severity must be thoroughly analyzed and controlled. For this reason, and in line with Cartín-Rojas et al. [15], hazards with severity = 5 were considered significant, even if their Criticality Index (CI) did not reach the threshold value. The most effective control measures for these hazards, within a HACCP framework, are stricter PRPs or CCPs, which involve more intensive monitoring and verification activities. This criterion may reduce the relative influence of the detection parameter in determining hazard significance. However, the detection parameter remains valuable for assessing the effectiveness of control measures and supporting their validation within the HACCP system, particularly for hazards with lower severity or borderline classification. Importantly, this severity-driven approach contributes to the consistency observed between the FMEA and the 4 × 4 matrix methods. While the 4 × 4 matrix inherently prioritizes hazards with high severity even at moderate probability levels, the inclusion of high-severity hazards in the FMEA classification leads to the identification of comparable significant hazards in both methods. Therefore, exceeding the severity score reinforces the convergence between both methodologies. Nevertheless, the FMEA method still provides added value by incorporating the detection parameter, offering a more detailed analysis of control measure performance and supporting continuous improvement within the HACCP framework.

After carrying out the risk assessment of the table olive production process using both matrices, it can be concluded that the same significant hazards have been identified. This result shows that the two matrices are valid for carrying out the risk assessment in the table olive production process.

#### 3.1.3. CCPs and Stricter PRP Definition

The final goal in any food safety risk assessment is to determine which stages must be strictly controlled. CCPs are the specific stages in the production process where targeted actions can be taken to prevent, eliminate, or reduce a significant food safety risk to an acceptable level [72]. To determine which production stages must be considered as CCPs, it is recommended to use the decision tree outlined by the Codex Alimentarius [39].

In the case of this specific product of Spanish-style olive processing, given that both studied risk matrices identified the same stages with significant hazards, it can be inferred that both tools equally provide an effective framework for risk assessment and, ultimately, for determining CCPs throughout table olive production. Therefore, all significant hazards identified previously in the risk assessment by both methods were subjected to this useful tool. A representative subset of the results is shown in Table 3.

As is shown in Table 3, the stages that were identified as PCC after applying both risk assessment matrices are: (i) biological risk in fermentation; (ii) biological risk in storage in fermentation vessels; (iii) biological risk in fruit conditioning after storage; (iv) biological risk in fruit reception and discharge; (v) physical risk in metal detection; (vi) biological risk in brine addition; (vii) biological risk in container sealing; (viii) biological risk in pasteurization and (ix) physical risk in x-ray detection.

First, the Quality Management Guide for the Table Olive Industry [13] presents storage after fermentation as a CCP in the production process for obtaining table olives to comprehensively control undesirable pathogenic or non-pathogenic microorganisms and/or toxins. Likewise, Valero et al. [7] presented an example of a CCP as the monitoring of pH level during olive fermentation. Finally, pasteurization is also reported as a CCP by Suherman et al. [73] because it is a control measure specifically designed to reduce the microbiological hazard of the product. In fact, in the table olive production process, pasteurization of packed olives is the last stage where the microbiological hazards can be controlled. Hence, pasteurization must be considered as a CCP in this process. The latest version of the Codex Alimentarius decision tree proposes considering all the control measures that act in combination against the same hazard as CCPs [39]. In this sense, the control of pH and NaCl concentration in fermentation, storage in fermentation vessels, fruit conditioning after storage, fruit reception and discharge, the NaCl concentration in brine addition, the container sealing and pasteurization act together against a common hazard: the possible growth or survival of pathogenic microorganisms. Hence, the control measures of all these stages should be categorized as CCPs.

Respecting physical hazard detection equipment, organizations usually include checkpoints for metal and/or non-metal detection as a CCP in the final part of their production processes to ensure food safety. For instance, Chen et al. [74] contemplated if the control measure metal detection could be a CCP. This author considered the need to identify specific CCPs related to the physical hazards present in powder and liquid sauce due to the high risk to consumer health and safety.

On the other hand, significant hazards that are not addressed through CCPs still require effective control measures. In this context, the Codex Alimentarius [39] decision tree acknowledges that certain significant hazards may be managed through prerequisite programs that demand more rigorous oversight than standard ones—such as enhanced monitoring and documentation. In this work, these are referred to as stricter PRPs. Accordingly, all stricter PRPs identified are shown in the Supplementary Data, Table S5. Nevertheless, Table 3 shows a representative subset of stricter PRPs identified in some stages of the process. The following PRPs were identified as stricter programs: (i) supplier approval plan to reduce the risk associated with olives, packaging materials and NaOH and NaCl; (ii) preventive maintenance to control physical hazards throughout the entire process; (iii) water control plan to mitigate the chemical and biological hazards present in water, and (iv) good manufacturing practices to avoid the breakage of glass containers in the final stages of the process.

Concerning supplier controls, Onyeaka et al. [44] described the importance of this PRP to ensure the safety of raw materials, ingredients, and auxiliary substances. This same author also presents the maintenance programs for facilities and equipment and good manufacturing practices to reduce the risk of foreign bodies in the final product as essential in production processes. Additionally, implementing a comprehensive water management plan is a key strategy to mitigate water-related risks, as highlighted by Allende et al. [75] and Chen et al. [50].

### 3.2. Californian-Style Processing

#### 3.2.1. Hazard Identification

Californian-style olive processing has similar points to Spanish-style, but with keys differences in three stages (Supplementary Data, Section S3). All identified hazards are the same for this process in stages 1 to 10, 17 to 34, and 37. The physical hazard of glass presence is eliminated in stages 36 and 38 to 41, due to the use of cans for packaging of this product, so these stages do not represent any safety hazards in processing. A detailed identification of safety hazards was made in these three different stages for Californian-style olive processing, and results are shown in Table 4.

In stages I and II, previously identified hazards in the storage stage, such as foreign matter, heavy metals in water, and pathogenic microbial growth during green olive storage, are similar in Californian-style processing. However, the introduction of air blowing in these two phases represents an additional safety hazard. If the quality of the air used is not adequately monitored and filtered, it may serve as a vector for airborne contaminants, including microbial spores, volatile chemical residues, and particulate matter, thereby compromising product safety and process hygiene [76]. Ensuring air quality through validated filtration and monitoring systems is essential to mitigate these emerging risks [77].

In stage II, the use of ferrous gluconate (E-579) as a food additive and iron supplement is widely accepted in the food industry due to its bioavailability and functional properties. However, its inclusion in any food processing must be carefully regulated to ensure consumer safety. According to the Joint FAO/WHO Expert Committee on Food Additives (JECFA) [78], the provisional maximum tolerable daily intake (PMTDI) for total iron is set at 0.8 mg/kg body weight, and no specific Acceptable Daily Intake (ADI) has been established for ferrous gluconate, as its contribution to total dietary gluconic acid is considered negligible when used appropriately. In the United States, the FDA classifies ferrous gluconate as Generally Recognized As Safe (GRAS) when used in accordance with good manufacturing practices [79]. Nevertheless, in the case of the European Union established an individual restriction for black olives darkened by oxidation of 150 ppm Commission Regulation (EU) No 1129/2011 of Food Additives [80]. Thus, this level must be controlled in the process and must be taken in account as a food safety chemical risk.

On the other hand, the fact that pH on these products could be relatively high (pH > 4.5), botulism, caused by the growth of *Clostridium botulinum*, represents the most significant biological hazard associated with this product and requires a sterilization phase to guarantee the safety of these products. Several outbreaks linked to homemade table olives, along with product recalls due to suspected contamination, have been reported. Some major illness outbreaks associated with botulism in table olives due to improper practices in industry include a total of 29 deaths in the USA for improper sterilization of black oxidized olives [18], or two cases and one death due to incorrect manufacturing of olives stuffed with almonds in Finland [24]. Notably, these incidents have been frequently associated with artisanal production practices or improper storage and treatment conditions in the industry, particularly when the pH of the product is not correct or thermal treatment is not adequate.

#### 3.2.2. Risk Matrix Comparison

After using both risk matrices (4 × 4 and FMEA) for specific stages of pitted black Manzanilla Californian-style olives packed in cans, the results are shown in the Supplementary Data, Table S6. Risks about foreign matter, heavy metals in water, and pathogenic microbial growth in stages I and II were evaluated with the same results for significance in similar stages of Spanish-style olive processing; no difference between each method was detected. It is notable that adding air blowing at this stage does not modify the significance of the biological risk but must be taken into account in the next steps of the risk analysis and in the establishment of control measures.

In stage II it is notable that the control of iron salt levels was a new significant chemical risk considered by both method, which is logical given the existence of legal limits established in Europe, the exceedance of which could render the product legally unsafe by the Commission Regulation (EU) No 1129/2011 of Food Additives [80]. Thus, an additional and specific control measure of blowing air quality must be taken into account in the next steps of HACCP (CCP or stricter PRP) for biological risk.

In stage III, the survival of thermoresistant spore-forming bacteria, particularly *Clostridium botulinum*, was consistently identified as a significant hazard by both the FMEA and 4 × 4 risk assessment matrices. This methodological convergence highlights the robustness of both tools in detecting high-severity biological risks associated with insufficient thermal processing. The FMEA matrix emphasized this hazard mostly due to its high severity, while the 4 × 4 matrix reinforced its criticality by combining this with the possible probability of inadequate sterilization conditions having severe consequences for consumer health. The alignment between both approaches validates the prioritization of this hazard and supports the implementation of stringent thermal treatments and pH control. This is especially relevant given the historical association of botulism outbreaks with improperly processed oxidized black table olives [18].

#### 3.2.3. CCP and PRP Definition

Significant risks in specific stages of Californian-style black olive processing were analyzed by the decision tree outlined by the Codex Alimentarius [39]. Complete analysis is shown in the Supplementary Data, Table S7. As is shown in Table S7, the specific stages of Californian-style processing that were identified as PCC after applying both risk assessment matrices are: (i) biological risk in stage I, (ii) chemical risk (iron salt) in stage II, (iii) biological risk in stage II and (iv) biological risk in sterilization in stage III.

Regarding the chemical risk of excess iron salt, it is logical that this is considered to be a CCP, because there is a legal limit in Europe for olives, and monitoring the levels of ferrous gluconate in this process is essential to prevent excessive iron intake, which may lead to adverse health effects such as gastrointestinal distress or iron overload in sensitive populations [81]. Regarding the limits, although the European limit for black olives is 150 ppm, an operative limit of 110 ppm is established in this process to ensure that the regulatory threshold is never exceeded, thereby maintaining the product’s safety.

Regarding biological risks in stages I and II, as was mentioned before, the latest version of the Codex Alimentarius decision tree proposes considering CCPs to be all the control measures that act in combination against the same hazard [39]. In this context, pH and air quality controls in storage and oxidation act together with sterilization against a common hazard: the possible growth or survival of heat-resistant bacteria (sporulated). Hence, the control measures of these two stages should be categorized as CCPs as well as the sterilization phase. With respect to thermal treatment, this study agrees with the International Olive Council (IOC) in proposing the sterilization stage to be a CCP, with temperatures that achieve almost 121 °C and times above 15 min as control values, as outlined in the Quality Management Guide for the Table Olive Industry [13].

## 4. Conclusions

This study confirms that both risk assessment methodologies, the 4 × 4 risk matrix and FMEA, are effective and reliable for identifying significant hazards in table olive processing. Their application across Spanish-style and Californian-style production lines demonstrated strong alignment in hazard prioritization, validating their suitability within HACCP frameworks. The 4 × 4 matrix emerges as the most practical tool for early-stage HACCP development and for processes with extensive flow charts and limited historical data. Its simplicity facilitates rapid implementation and reduces subjectivity in decision-making. Conversely, the FMEA approach, enriched by the inclusion of detection probability, offers enhanced analytical depth and is particularly valuable for validating established systems or supporting continuous improvement initiatives.

Importantly, the study identifies the biological hazards associated with fermentation, storage, brine control, and pasteurization/sterilization as the most critical across both processing types. Physical hazards and chemical hazards—especially pesticide residues, heavy metals, mycotoxins, and chemical migration—require reinforced monitoring and well-structured PRPs. The results strongly support the synergistic application of CCPs and stricter PRPs, aligning with the updated Codex Alimentarius recommendations.

Overall, the findings contribute to enhancing scientific rigor in HACCP planning and support the modernization of sector-specific risk management guidelines. The adoption of standardized, validated risk matrices can improve transparency, reproducibility, and risk communication throughout the table olive industry.

Furthermore, this study provides one of the first structured comparative evaluations of commonly used risk assessment matrices within the same industrial HACCP framework, contributing practical guidance for their selection and application.

## Figures and Tables

**Table 1:** Representative subset of hazard identification and control measures in Spanish-style Manzanilla pitted green olives packed in glass.

Stage	Hazards	Control Measures Already Implemented
Olive receiving hopper	(P) Large debris, such as branches and leaves	Separation of large debris in receiving hoppers
	(C) Heavy metals, pesticides and mycotoxins	Supplier approval plan Reception plan
	(B) Microorganisms (*Escherichia coli*, *Salmonella*, *Clostridium*…)	
Reception of packaging and labeling materials	(P) Foreign bodies	Supplier approval plan Reception control plan
	(C) Chemical migration	
	(B) Microorganisms (bacteria, viruses, molds, and yeasts) and pests	
Washing (I)	(P) Foreign bodies	Preventative maintenance
	(C) Heavy metals	Water control plan
	(B) Microorganisms (*Escherichia coli*, *Clostridium perfringens*…)	Water control plan
Lye treatment	(P) Foreign bodies	Preventative maintenance
	(C) Equipment, chemicals, and remains of cleaning and disinfection products	Preventative maintenance Cleaning and disinfection plan
	(B) None	None
Fermentation	(P) Foreign bodies	Preventative maintenance
	(Q) Remains of cleaning and disinfection products	Cleaning and disinfection plan
	(B) Microorganisms (*Enterobacteriaceae*, *Clostridium*, *Pseudomonas*, *Staphylococcus*, etc.)	Control of pH, salt, and free and combined acidity Control of the absence of bad odors and flavors Removal of fermenting surface veils
Storage in fermentation vessels	(P) Foreign bodies	Preventative maintenance
	(Q) Remains of cleaning and disinfection products	Cleaning and disinfection plan
	(B) Microorganisms (*Propionibacteria*, aerobic fungi, *Clostridium*)	Control of pH (<4.3) and NaCl (>8%) Removal of fermenting surface veils
Fruit conditioning after storage	(P) Foreign bodies	Preventative maintenance
	(Q) Equipment, chemicals, and remains of cleaning and disinfection products	Preventative maintenance Cleaning and disinfection plan
	(B) Microorganisms (*Clostridium*, *Staphylococcus*, *Pseudomonas*, *Enterobacteriaceae*)	Control of pH (<4.3) and NaCl (4–5%)
Fruit reception and discharge	(P) Foreign bodies	Preventative maintenance
	(Q) Remains of cleaning and disinfection products	Cleaning and disinfection plan
	(B) Microorganisms (*Clostridium*, *Staphylococcus*, *Pseudomonas*, *Enterobacteriaceae*)	Control of pH and NaCl Cleaning and disinfection plan
Metal detection	(P) Foreign metal bodies	Metal detection
	(C) Remains of cleaning and disinfection products	Cleaning and disinfection plan
	(B) Microorganisms present on machinery (mainly mesophilic aerobes)	Cleaning and disinfection plan
Brine addition	(P) Foreign bodies	Preventative maintenance
	(C) Equipment, chemicals, and remains of cleaning and disinfection products	Preventative maintenance Cleaning and disinfection plan
	(B) Microorganisms present on machinery (mainly mesophilic aerobes) and brine	Cleaning and disinfection plan Control of the brine [NaCl (4–5%), pH (<4.3) and acidity (0.3–0.5%)]
Container sealing	(P) Foreign bodies	Preventative maintenance
	(C) Equipment, chemicals, and remains of cleaning and disinfection products	Preventative maintenance Cleaning and disinfection plan
	(B) Microorganisms	Control of hermetic sealing
Pasteurization	(P) None	None
	(C) None	None
	(B) Microorganisms (*Clostridium*, aerobic mesophiles, fungal spores)	Temperature: 70–85 °C Time: 5–20 min 15 PU units
X-ray detection	(P) Foreign bodies	X-ray detection
	(C) None	None
	(B) None	None
Labeling Palletizing Storage Shipping	(P) Glass	Good manufacturing practices
	(C) None	None
	(B) None	None

P = Physical hazard; C = Chemical hazard; B = Biological hazard.

**Table 2:** Representative subset of hazard risk analysis after applying 4 × 4 matrix and FMEA model in Spanish-style olive processing.

Stage	Hazard	Control Measures	4 × 4 Matrix Model				FMEA Model				
			Probability	Severity	Risk	SIG	Probability	Severity	Detection	CI	SIG
Olive receiving hopper	(C) Heavy metals, pesticides, and mycotoxins	Supplier approval plan Reception plan	3 Probably	B Severe, not imminent	3B Major	Yes	3 Occasional	5 Very severe	1 Existing detection measures	15	Yes S = 5
	(B) Microorganisms (*Escherichia coli*, *Salmonella*, *Clostridium*…)	Supplier approval plan Reception plan	2 Possible	A Severe	2A Major	Yes	3 Occasional	5 Very severe	1 Existing detection measures	15	Yes S = 5
Reception of packaging and labeling materials	(P) Foreign bodies	Supplier approval plan Reception control plan	2 Possible	A Severe	2A Major	Yes	3 Occasional	5 Very severe	1 Existing detection measures	15	Yes S = 5
	(C) Chemical migration	Supplier approval plan Reception control plan	2 Possible	B Severe, not imminent	2B Medium	Yes	2 Unlikely	5 Very severe	1 Existing detection measures	10	Yes S = 5
Reception of sodium hydroxide (NaOH) and salt (NaCl) reception	(C) Incorrect chemical	Supplier approval plan Reception control plan	1 Unlikely	A Severe	1A Medium	Yes	2 Unlikely	5 Very severe	1 Existing detection measures	10	Yes S = 5
Washing (I)	(P) Foreign bodies	Preventative maintenance	2 Possible	A Severe	2A Major	Yes	3 Occasional	5 Very severe	1 Existing detection measures	15	Yes S = 5
	(C) Heavy metals	Water control plan	2 Possible	B Severe not imminent	2B Medium	Yes	3 Occasional	5 Very severe	1 Existing detection measures	15	Yes S = 5
	(B) Microorganisms (*Escherichia coli*, *Clostridium perfringens*…)	Water control plan	2 Possible	A Severe	2A Major	Yes	3 Occasional	5 Very severe	1 Existing detection measures	15	Yes S = 5
Washing (II)	(P) Foreign bodies	Preventative maintenance	2 Possible	A Severe	2A Major	Yes	3 Occasional	5 Very severe	1 Existing detection measures	15	Yes S = 5
	(C) Heavy metals	Water control plan	2 Possible	B Severe not imminent	2B Medium	Yes	3 Occasional	5 Very severe	1 Existing detection measures	15	Yes S = 5
	(B) Microorganisms (*Escherichia coli*, *Clostridium perfringens*…)	Water control plan	2 Possible	A Severe	2A Major	Yes	3 Occasional	5 Very severe	1 Existing detection measures	15	Yes S = 5
Transport channels	(P) Foreign bodies	Preventative maintenance	2 Possible	A Severe	2A Major	Yes	3 Occasional	5 Very severe	1 Existing detection measures	15	Yes S = 5
	(C) Equipment chemicals and remains of cleaning and disinfection products Heavy metals in water	Preventative maintenance Cleaning and disinfection plan Water control plan	2 Possible	B Severe not imminent	2B Medium	Yes	3 Occasional	5 Very severe	1 Existing detection measures	15	Yes S = 5
	(B) Microorganisms	Cleaning and disinfection plan Water plan control	2 Possible	A Severe	2A Major	Yes	3 Occasional	5 Very severe	1 Existing detection measures	15	Yes S = 5
Flotation/vibrator tank	(P) Foreign bodies	Preventative maintenance	2 Possible	A Severe	2A Major	Yes	3 Occasional	5 Very severe	1 Existing detection measures	15	Yes S = 5
	(C) Equipment chemicals and remains of cleaning and disinfection products Heavy metals in water	Preventative maintenance Cleaning and disinfection plan Water control plan	2 Possible	B Severe, not imminent	2B Medium	Yes	3 Occasional	5 Very severe	1 Existing detection measures	15	Yes S = 5
	(B) Microorganisms	Cleaning and disinfection plan Water plan control	2 Possible	A Severe	2A Major	Yes	3 Occasional	5 Very severe	1 Existing detection measures	15	Yes S = 5
Metal detection	(P) Foreign metal bodies	Metal detection	3 Probably	A Severe	3A Major	Yes	3 Occasional	5 Very severe	1 Existing detection measures	15	Yes S = 5
Container sealing	(P) Foreign bodies	Preventative maintenance	2 Possible	A Severe	2A Major	Yes	3 Occasional	5 Very severe	1 Existing detection measures	15	Yes S = 5
	(B) Microorganisms	Control of hermetic sealing	2 Minor	A Severe	2A Major	Yes	3 Occasional	5 Very severe	1 Existing detection measures	15	Yes S = 5
Pasteurization	(B) Microorganisms (Clostridium, aerobic mesophiles, fungal spores)	Temperature: 70–85 °C Time: 5–20 min 15 PU units	2 Possible	A Severe	2A Major	Yes	3 Occasional	5 Very severe	1 Existing detection measures	15	Yes S = 5
Container drying	(P) Glass splinter	Good manufacturing practices	2 Possible	A Severe	2A Major	Yes	3 Occasional	5 Very severe	1 Existing detection measures	15	Yes S = 5
X-ray detection	(P) Foreign bodies	X-ray detection	3 Probably	A Severe	3A Major	Yes	3 Occasional	5 Very severe	1 Existing detection measures	15	Yes S = 5
Labelling	(P) Glass splinter	Good manufacturing practices	3 Probably	A Severe	3A Major	Yes	3 Occasional	5 Very severe	5 There are no detection measures	75	Yes

**Table 3:** Representative subset of CCPs and stricter PRPs determined in pitted Manzanilla green Spanish-style olives packed in glass.

Stage/Hazard	Risk 4 × 4 Matrix	Risk FMEA Method *	Q1	Q2	Q3	Q4	CCP/Stricter PRP
Olive receiving hopper (C) Heavy metals, pesticides and mycotoxins	Major	CI = 15 S = 5	Yes (SAP, RP)	-	-	-	Stricter PRP
Olive receiving hopper (B) Microorganisms (*Escherichia coli*, *Salmonella*, *Clostridium*…)	Major	CI = 15 S = 5	Yes (SAP, RP)	-	-	-	Stricter PRP
Washing (II) (C) Heavy metals	Medium	CI = 15 S = 5	Yes (WCP)	-	-	-	Stricter PRP
Washing (II) Microorganisms (*Escherichia coli*, *Clostridium perfringens*…)	Major	CI = 15 S = 5	Yes (WCP)	-	-	-	Stricter PRP
Fermentation (B) Microorganisms (Enterobacteriaceae, Clostridium, Pseudomonas, Staphylococcus, etc.)	Major	CI = 15 S = 5	No	Yes	Yes (P)	-	** CCP
Storage in fermentation vessels (P) Foreign bodies	Major	CI = 15 S = 5	Yes (PM)	-	-	-	Stricter PRP
Storage in fermentation vessels (B) Microorganisms (Enterobacteriaceae, Clostridium, Pseudomonas, Staphylococcus, etc.)	Major	CI = 15 S = 5	No	Yes	Yes (P)	-	** CCP
Fruit conditioning after storage (P) Foreign bodies	Major	CI = 15 S = 5	Yes (PM)	-	-	-	Stricter PRP
Fruit conditioning after storage (B) Microorganisms (Enterobacteriaceae, Clostridium, Pseudomonas, Staphylococcus, etc.)	Major	CI = 15 S = 5	No	Yes	Yes (P)	-	** CCP
Fruit reception and discharge (P) Foreign bodies	Major	CI = 15 S = 5	Yes (PM)	-	-	-	Stricter PRP
Fruit reception and discharge (B) Microorganisms (Enterobacteriaceae, Clostridium, Pseudomonas, Staphylococcus, etc.)	Major	CI = 15 S = 5	No	Yes	Yes (P)	-	** CCP
Metal detection (P) Foreign metal bodies	Major	CI = 15 S = 5	No	Yes	No	Yes	CCP
Brine addition Microorganisms	Major	CI = 15 S = 5	No	Yes	Yes (P)	-	** CCP
Container sealing (B) Microorganisms	Major	CI = 15 S = 5	No	Yes	Yes (P)	-	** CCP
Pasteurization (B) Microorganisms (Clostridium, aerobic mesophiles, fungal spores)	Major	CI = 15 S = 5	No	Yes	No	Yes	CCP
X-ray detection (P) Foreign bodies	Major	CI = 15 S = 5	No	Yes	No	Yes	CCP
Labelling (P) Glass splinter	Major	CI = 15 S = 5	Yes (GMP)	-	-	-	Stricter PRP

P = Physical hazard; C = Chemical hazard; B = Biological hazard; SAP = Supplier Approval Plan; RP = Reception Plan; GMP = Good Manufacturing Practices; WCP = Water Control Plan; PM = Preventive Maintenance. * Risk: CI = Criticality Index; S = Severity. ** Consider whether the control measure at this step works in combination with a control measure at another step to control the same hazard, in which case both steps should be considered as CCPs.

**Table 4:** Hazard identification and control measures already implemented in specific stages of Californian-style olive processing.

Stage	Hazards	Control Measures Already Implemented
I.-Storage in fiberglass tanks with acidified brine and air blowing	(P) Foreign bodies	Preventative maintenance
	(C) Heavy metals in water	Water control plan
	(B) Microorganisms (*Escherichia*, *Clostridium*, molds, etc.)	Control of pH (pH < 4.3), free acidity (>1%), air blowing control.
II.-Oxidation, lye treatment, and color fixation in aeration tanks	(P) Foreign bodies	Preventative maintenance
	(C) Residual iron salts (ferrous gluconate) in excessive amounts	Iron salt control (110 ppm)
	(B) Microorganisms (*Escherichia*, *Clostridium*, etc.)	Absence of bad odors and flavors.
III.-Sterilization	(P) N/A	---
	(C) N/A	---
	(B) Survival of heat-resistant bacteria (sporulated) if the process is inadequate	Temperature: 121 °C Time: >15 min

P = Physical hazard; C = Chemical hazard; B = Biological hazard.

## Data Availability

The original contributions presented in the study are included in the article/Supplementary Material, further inquiries can be directed to the corresponding author.

## Supplementary Materials

The following supporting information can be downloaded at https://www.mdpi.com/article/10.3390/foods15122153/s1, Figure S1. Process flow chart for green pitted Sevillian-style olives; Figure S2. Process flow chart for black pitted Californian-style olives; Table S1. 4 × 4 risk matrix for hazards; Table S2. Risk matrix based on FMEA; Table S3. Hazard identification and control measures already implemented in Spanish-style olive processing; Table S4. Hazard analysis after applying 4 × 4 matrix and FMEA model in Spanish-style olive processing; Table S5. CCP and stricter PRP determination after applying 4 × 4 matrix and FMEA model in Spanish-style olive processing; Table S6. Hazard analysis after applying 4 × 4 matrix and FMEA model in in specific stages of Californian-style black olive processing; Table S7. CCP and stricter PRP determination after applying 4 × 4 matrix and FMEA model in specific stages of Californian-style black olive processing.
