# Supplementary material for: Enhancing HACCP Decisions: A Comparative Risk Assessment for Table Olive Processing
Source: Foods. 2026 Jun 14;15(12):2153. doi: 10.3390/foods15122153 (PMC13298240; doi:10.3390/foods15122153)
Supplement: Supplementary file 1 [file foods-15-02153-s001.zip › Figures S1 and S2 format PPT.pptx]

## Slide 1
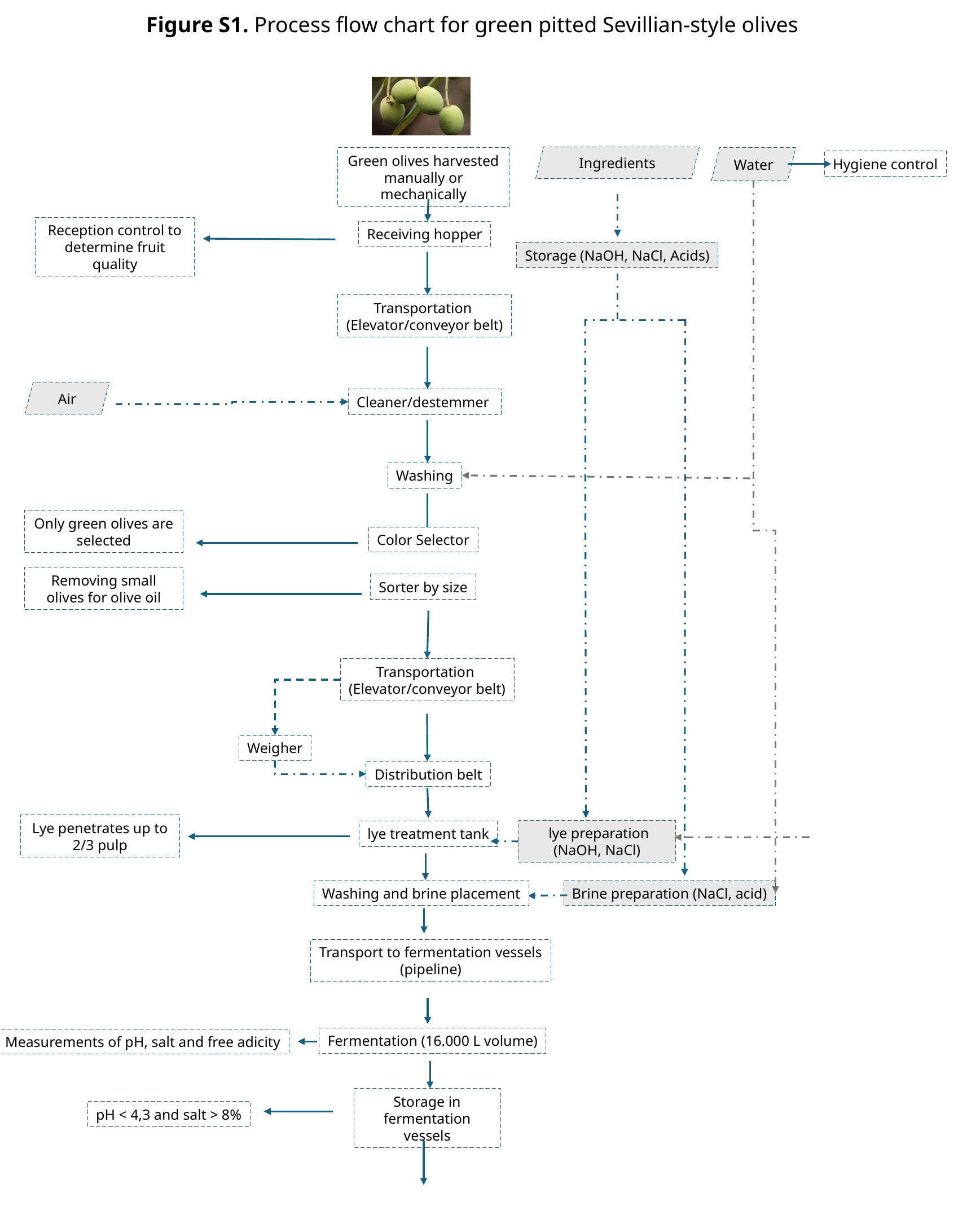

Figure S1. Process flow chart for green pitted Sevillian-style olives
Ingredients
Water
Green olives harvested manually or mechanically
Receiving hopper
Storage (NaOH, NaCl, Acids)
Transportation
(Elevator/conveyor belt)
Air
Cleaner/destemmer
Washing
Sorter by size
Transportation
(Elevator/conveyor belt)
Weigher
Distribution belt
 lye preparation (NaOH, NaCl)
lye treatment tank
Brine preparation (NaCl, acid)
Transport to fermentation vessels
(pipeline)
Fermentation (16.000 L volume)
Storage in fermentation vessels
Hygiene control
Reception control to determine fruit quality
Only green olives are selected
Color Selector
Removing small olives for olive oil
Lye penetrates up to 2/3 pulp
Washing and brine placement
Measurements of pH, salt and free adicity
pH < 4,3 and salt > 8%

## Slide 2
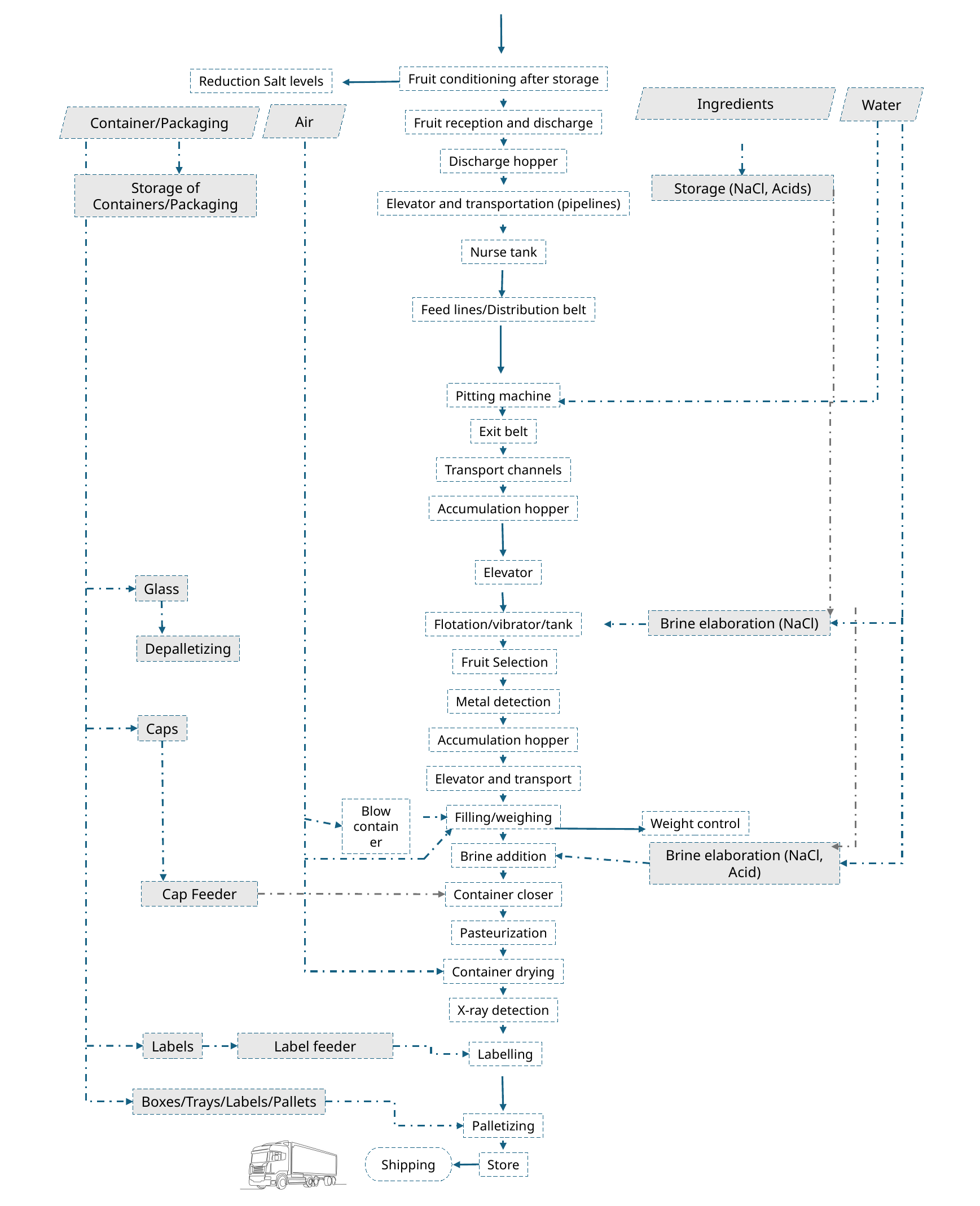

Fruit conditioning after storage
Reduction Salt levels
Water
Ingredients
Air
Container/Packaging
Fruit reception and discharge
Discharge hopper
Storage of Containers/Packaging
Storage (NaCl, Acids)
Elevator and transportation (pipelines)
Nurse tank
Feed lines/Distribution belt
Pitting machine
Exit belt
Transport channels
Accumulation hopper
Elevator
Glass
Brine elaboration (NaCl)
Flotation/vibrator/tank
Depalletizing
Fruit Selection
Metal detection
Caps
Accumulation hopper
Elevator and transport
Blow
container
Filling/weighing
Weight control
Brine elaboration (NaCl, Acid)
Brine addition
Cap Feeder
Container closer
Pasteurization
Container drying
X-ray detection
Labels
Label feeder
Labelling
Boxes/Trays/Labels/Pallets
Palletizing
Shipping
Store

## Slide 3
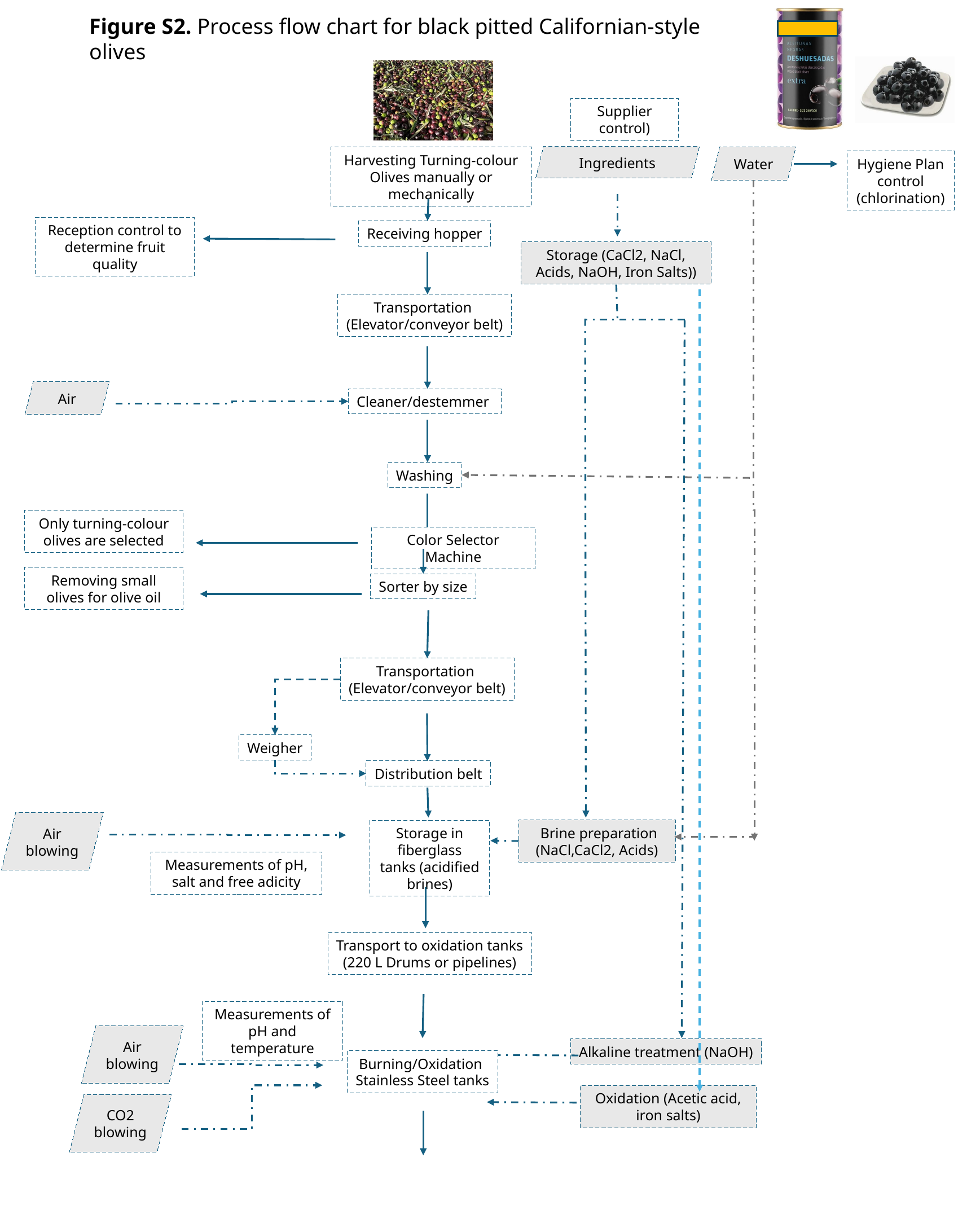

Figure S2. Process flow chart for black pitted Californian-style olives
Supplier control)
Ingredients
Water
Harvesting Turning-colour Olives manually or mechanically
Receiving hopper
Storage (CaCl2, NaCl, Acids, NaOH, Iron Salts))
Transportation
(Elevator/conveyor belt)
Air
Cleaner/destemmer
Washing
Sorter by size
Transportation
(Elevator/conveyor belt)
Weigher
Distribution belt
 Brine preparation (NaCl,CaCl2, Acids)
Storage in fiberglass tanks (acidified brines)
Transport to oxidation tanks
(220 L Drums or pipelines)
Alkaline treatment (NaOH)
Hygiene Plan control (chlorination)
Reception control to determine fruit quality
Only turning-colour olives are selected
Color Selector Machine
Removing small olives for olive oil
Air blowing
Measurements of pH, salt and free adicity
Measurements of pH and temperature
Air blowing
Burning/Oxidation
Stainless Steel tanks
Oxidation (Acetic acid, iron salts)
CO2 blowing

## Slide 4
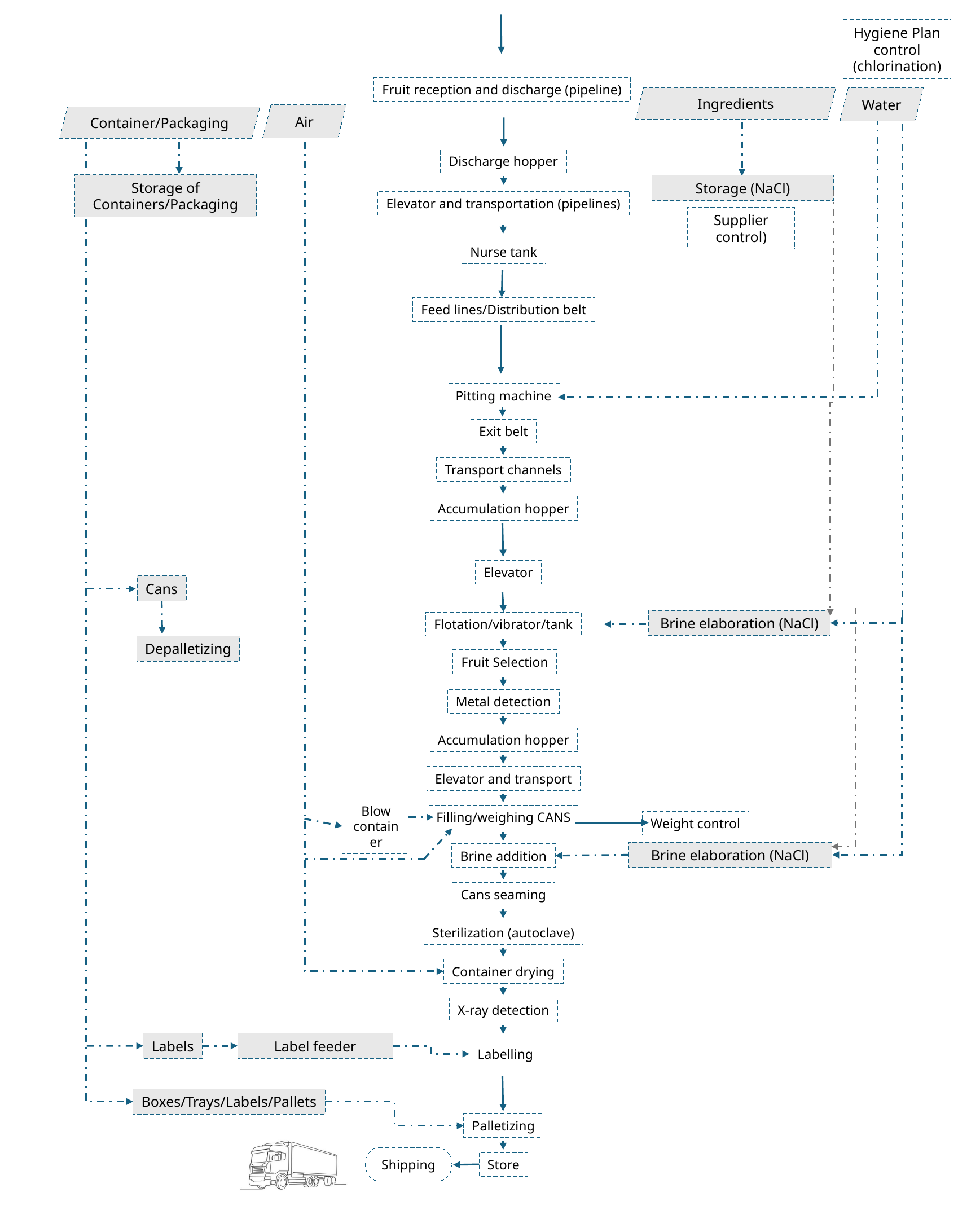

Hygiene Plan control (chlorination)
Fruit reception and discharge (pipeline)
Water
Ingredients
Air
Container/Packaging
Discharge hopper
Storage of Containers/Packaging
Storage (NaCl)
Elevator and transportation (pipelines)
Supplier control)
Nurse tank
Feed lines/Distribution belt
Pitting machine
Exit belt
Transport channels
Accumulation hopper
Elevator
Cans
Brine elaboration (NaCl)
Flotation/vibrator/tank
Depalletizing
Fruit Selection
Metal detection
Accumulation hopper
Elevator and transport
Blow
container
Filling/weighing CANS
Weight control
Brine elaboration (NaCl)
Brine addition
Cans seaming
Sterilization (autoclave)
Container drying
X-ray detection
Labels
Label feeder
Labelling
Boxes/Trays/Labels/Pallets
Palletizing
Shipping
Store
