# Supplementary material for: Enhancing HACCP Decisions: A Comparative Risk Assessment for Table Olive Processing
Source: Foods. 2026 Jun 14;15(12):2153. doi: 10.3390/foods15122153 (PMC13298240; doi:10.3390/foods15122153)
Supplement: Supplementary file 1 [file foods-15-02153-s001.zip › foods-4334302-Supplementary Materials.pdf]

foods

Supplementary Materials

Enhancing HACCP decisions: A comparative risk assessment for table olive processing

Cristina Campanero Pintado[1], Kharla Andreina Segovia-Bravo[1], Antonio Benítez Cabello[2], Francisco Noé Arroyo-López[2] and Efrén Pérez-Santín[1*]

[1] Escuela Superior de Ingeniería y Tecnología (ESIT), Universidad Internacional de La Rioja (UNIR), Avenida de la Paz, 137, 26006 Logroño, Spain
[2] Instituto de la Grasa. Consejo Superior de Investigaciones Científicas. Campus Universitario Pablo de Olavide. Edificio 46. Carretera de Utrera, km 1. 41013 Seville, Spain
* Correspondence: efren.perez@unir.net

Table of Contents

S1. Description of raw and auxiliar materials

S2. Description of each stage in Spanish-style olive processing

S3. Description of each stage in black pitted Californian-style olive processing

Figure S1. Process flow chart for green pitted Sevillian-style olives

Figure S2. Process flow chart for black pitted Californian-style olives

Table S1. 4x4 risk matrix for hazards

Table S2. Risk matrix based on FMEA

Table S3. Hazard identification and control measures already implemented in Spanish-style olive processing

Table S4. Hazard analysis after applying 4x4 matrix and FMEA model in Spanish-style olive processing

Table S5. CCP and stricter PRP determination after applying 4x4 matrix and FMEA model in Spanish-style olive processing

Table S6. Hazard analysis after applying 4x4 matrix and FMEA model in in specific stages of Californian-style black olive processing

Table S7. CCP and stricter PRP determination after applying 4x4 matrix and FMEA model in specific stages of Californian-style black olive processing

 https://doi.org/10.3390/foods15122153

S1. Description of raw and auxiliar materials

Olives*:* Manzanilla variety harvested at the green to yellow-green ripening stage for Spanish-style and turning-colour for Californian-style.

Glass jars and metal caps*:* glass containers employed for packaging Spanish-style olives are made from soda-lime glass. This glass is non-reactive with brine solutions, ensuring that no undesirable interactions occur between the packaging and the olives. It also provides an effective barrier against oxygen and moisture, contributing to extended shelf life and microbial stability. Glass jars are sealed with metal caps lined with food-grade polymer gaskets to ensure hermetic closure and prevent contamination. The packaging is designed to withstand thermal processing, such as pasteurization, without compromising structural integrity.

Cans*:* Metal cans of 425ml are used for packaging black Californian-style olives. These are received as part of the packaging supply chain. These cans arrive from one specialized manufacturer and are delivered in bulk, often stacked on pallets and protected with shrink wrap or cardboard to prevent contamination and physical damage during transport. Cans are made with a food-grade coating to prevent interaction between the metal and the brine or olives.

Acetic, citric, lactic and chlorohydric acids*:* Acids food-grade standards are used as an acidifying agent in the process. They are received in high-density polyethylene (HDPE) containers of 1,000 liters with metal cages for protection and easy handling.

Iron salt*:* Powdered ferrous gluconate ($FeC_{12}H_{22}O_{14}$) is received, packed in sealed bags to prevent moisture absorption and contamination, and stored in a dry temperature-controlled area.

Calcium chloride*:* $CaCl_2$ is received in flakes delivered in sealed plastic bags and stored in a dry temperature-controlled area.

CO_2*:* CO_2 used as food-grade, ensuring it meets purity standards for safe use in food processing environments. It is received in bulk tanks.

Labels*:* labels are supplied in sealed rolls, with an external reference sample provided for visual inspection and verification. They are manufactured using inks and adhesives that meet food safety standards, ensuring resistance to humidity and temperature fluctuations encountered during storage and transportation. The label material typically consists of paper laminated with a protective polymer film to enhance durability and maintain print clarity.

Sodium hydroxide (NaOH): Sodium hydroxide (NaOH) is received in food-grade concentrated aqueous solution. This material is supplied in bulk. The solution is transferred through closed systems into dedicated storage tanks.

Brine: the brine, composed of sodium chloride (NaCl) dissolved in potable water (concentration between 6% and 10% w/v), is received as a pre-formulated bulk solution by food-grade tanker trucks.

Pallets*:* The pallets are received in standardized formats (EUR-pallet) with dimensions of 1200 mm × 1000 mm. They are made of heat-treated wood (HT), compliant with ISPM 15 regulations for international phytosanitary standards.

https://doi.org/10.3390/xxxxx

S2. Description of each stage in Spanish-style olive processing

1. **Olive receiving hopper:** the olives are transported in bulk trucks or open crates. Once they arrive at the processing plant, an inspection of the transport medium is carried out: registration of the certificate or declaration of the previous cargo and the cleaning performed, especially for bulk or container transports that may be used for other products. Data is recorded: entry date, owner, weight, type of olives, variety, average ripeness (particularly for green olives), average fruit size, proportion of unusable fruits, whether the olives are wet or dry, wrinkled or turgid, fruit damage, presence of foreign materials, etc. Additionally, collection date, treatment certificates provided by the grower, and the cultivation system (dryland or irrigated farming) are checked and recorded. The olives are then unloaded into receiving hoppers, where a preliminary separation of large debris such as branches and leaves takes place. At this stage, they are weighed, and an initial sampling may be performed to evaluate quality. The hopper allows handling large volumes and temporarily stores them before processing begins.
2. **Reception of packaging and labelling materials**. Packaging glass jars are received under controlled conditions to ensure compliance with food safety standards. Labelling materials arrive in protected roll formats, each featuring an external sample label for visual verification and batch validation.
   The safety and quality assurance of all materials intended to come into contact with food is governed by the supplier approval protocol. This protocol mandates adherence to relevant legal frameworks, including good manufacturing practices, and requires the provision of a declaration of conformity certifying that the materials are suitable for food contact applications.
3. **Chemicals reception**: NaOH and acids are received and subjected to quality control checks, including concentration verification (30–50% w/w for liquid NaOH), pH measurement, and purity assessment to ensure compliance with food-grade specifications.
4. **Brine reception:** the brine is received as a pre-formulated bulk solution by food-grade tanker trucks. Upon arrival, the brine is transferred through closed systems to dedicated storage tanks equipped with agitation systems to maintain homogeneity and prevent salt precipitation. Quality control procedures are implemented to verify salt concentration (between 20% and 25% w/v), microbiological safety, pH, and conductivity, and compliance with food quality standards.
5. **Transportation elevator/conveyor belt:** olives are transported using elevators or conveyor belts. These belts are designed to move large quantities of olives smoothly, minimizing the risk of mechanical damage. Some belts include sensors to control the flow and avoid jams during the transfer to the next stage.
6. **Cleaner/destemmer:** in the cleaner/destemmer, olives pass through vibrating rollers that remove branches, leaves, and other small debris. This ensures that only olives continue to the washing process, reducing the amount of foreign material and improving the efficiency of subsequent steps.
7. **Washing I:** the olives are washed with potable water of controlled quality, usually using rotary drum washers or continuous flow washers. Soft water is typically used to avoid mineral deposits that might affect the process. The water is frequently replaced to ensure proper cleaning of the olives.
8. **Sorter by size:** olives are sorted by size using vibrating screens or automatic graders. This ensures they are grouped by uniform sizes, facilitating homogeneous treatment in subsequent stages. Typical diameters range from 14 mm to 24 mm (60 – 410 fruits/kg), classified according to industry standards.

9. **Colour selector:** olives pass through an optical and automated selection system, where cameras and sensors identify and remove olives with undesirable colours (black or colour-turning olives) or damage. Only those with a uniform green hue are selected for further processing, ensuring the right visual quality and ripeness level.
10. **Transportation (elevator/conveyor belt):** the classified olives are transported again by belts or elevators to the chemical treatment tanks, ensuring a continuous and efficient process flow.
11. **Lye treatment tank:** the olives are treated with a sodium hydroxide (NaOH) solution at a concentration of 1.5% to 2.5% (15 to 25 g/L) to remove bitterness. Sometimes, salt (2-3%) is also added to the lye solution. The lye penetrates about two-thirds (2/3) of the olive pulp, breaking down phenolic compounds like oleuropein. The soaking time varies from 8 to 10 hours, depending on the size and variety of the olives. Agitation systems are sometimes used to ensure an even distribution of the lye. This treatment also facilitates subsequent lactic fermentation and helps develop the unique organoleptic characteristics of the product.
12. **Washing II and brine placement:** in the same tank, the lye solution is then replaced by tap water to remove excess NaOH from fruits. The process of washing can extend from 6 to 12h. After this time, washing liquid is replaced by brine prepared with sodium chloride (NaCl) at concentrations of 8% to 10% (80 to 100 g/L) and usually acidified with HCl to neutralize remnants of bleach. Olives can stay up to 12 h in brine before transport to fermentation vessels. There is a dosage control to guarantee the correct NaCl concentration.
13. **Transport to fermentation vessels (pipeline):** after washing treatment, olives are transported through pipelines to fermentation tanks. The pipes are designed to minimize air contact, reducing oxidation risk and maintaining product quality.
14. **Fermentation:** the fermentation process, lasting several weeks or months, is mainly carried out by lactic acid bacteria, producing lactic acid that lowers the brine's pH to a range of 3.8 to 4.2. This acidic environment helps preserve the product and develop its characteristic flavours. The fermentation is carried out in fermentation vessels of 16.000L of volume (9.500 kg of fruits + 5500 L of brine) that are usually underground. During fermentation, pH, salt, free and combined acidity are monitored, as well as the absence of bad odours or flavours.
15. **Storage in fermentation vessels:** after fermentation, the olives remain in the same tanks for storage for long periods (6 – 12 months) of time until demand. Periodic checks are conducted to verify the stability of pH and salt concentration, typically maintained above 8% NaCl. This adjustment prevents the growth of undesirable microorganisms and ensures product quality until packaging.
16. **Fruit conditioning after storage:** after fermentation and storage, and when olive is in demand for packaging, olives go through a conditioning stage to prepare them for final processing. This may include brine adjustments to balance flavour and stabilize pH (pH around 4.3) and salt (4-5% NaCl). Conditioning helps to standardize product characteristics before packaging.
17. **Fruit reception and discharge:** the conditioned olives are received from storage tanks and discharged into transport systems for final processing. The quality of the batch is checked with quick pH and salinity tests to ensure they meet established parameters.
18. **Discharge hopper:** in this stage, olives are discharged into hoppers. These temporary hoppers regulate the flow to subsequent machines in the process and facilitate continuous feeding, avoiding interruptions in the production line.

19. **Elevator and transportation (pipelines):** the olives are transported through elevators or pipelines to the next processing step. The use of pipelines allows for gentle handling, minimizing physical damage. The transport can be carried out with brine or water to facilitate movement.
20. **Nurse tank:** in the nurse tank, the olives are temporarily accumulated to be evenly distributed to different processing lines. This ensures a constant flow and prevents a lack of raw material in subsequent stages.
21. **Feed lines/distribution belt:** from the nurse tank, olives are distributed via conveyor belts or feed lines to pitting or stuffing machines. These belts are equipped with flow control systems to maintain a continuous and regular feed.
22. **Pitting machine:** olives pass through a pitting machine, which uses punches to perforate and extract the pit from each fruit. The precision of these machines is crucial to avoid pulp damage and maintain a uniform appearance. The machine's speed varies based on the line capacity, handling from 300 to 500 olives per minute.
23. **Exit belt:** pitted olives exit the machine via an exit belt, where they are visually inspected to remove any damaged or poorly pitted fruit.
24. **Transport channels:** the olives are transported via water channels or conveyor belts to the next stage. Water channels help clean any residue generated during pitting.
25. **Accumulation hopper:** the pitted olives are temporarily stored in an accumulation hopper before moving to the next phase of sorting and selection. This hopper helps regulate the flow towards the classification systems.
26. **Elevator:** from the accumulation hopper, the olives are lifted again to proceed to the flotation, selection, or classification process according to the production line design.
27. **Flotation/vibrator tank:** the olives undergo a flotation process in tanks filled with saltwater or solutions with adjusted density. This step separates olives based on their quality and condition (e.g., defective olives tend to float and can be removed). Vibrators help dislodge any small particles clinging to the olives.
28. **Fruit selection:** at this stage, a manual or automatic selection of the olives takes place. Automatic systems can use optical sensors to detect defects, blemishes, or irregularities, removing olives that do not meet quality standards.
29. **Metal detection:** before packaging, the olives pass through metal detectors to ensure they are free from metallic contaminants, safeguarding consumer health and preventing food safety issues.
30. **Accumulation hopper:** selected olives are accumulated again in a hopper before being directed to the packaging process, ensuring a continuous flow towards the filling machines.
31. **Elevator and transport:** from the accumulation hopper, olives are lifted and transported via belts to the filling stations. This transport is carried out gently to avoid damaging the product.
32. **Filling/weighing:** at the filling station, the olives are automatically dispensed into containers (glass jars). The filling machines are calibrated to dispense the exact quantity, ensuring uniform and precise weights.
33. **Brine addition:** after filling, brine is added to the container to preserve the olives. The brine typically has a concentration in the equilibrium of 4% to 5% NaCl, pH 4,0 – 4,3 and acidity between 0,3- 0,5%, adjusted to ensure product preservation and maintain its characteristic flavour.
34. **Container sealing:** the containers are automatically sealed using hermetic sealing systems. Twist-off caps are used for glass jars. There is a control to ensure the hermetic sealing.
35. **Pasteurization:** the sealed containers pass through a pasteurization tunnel, where they are exposed to temperatures ranging from 70°C to 85°C for 5 to 20 minutes. This process eliminates pathogenic microorganisms and extends the

product's shelf life. However, pasteurization does not eliminate resistant forms such as spores. The pasteurization process is applied until a minimum of 15 PU units is obtained.

36. **Container drying:** the containers are dried in a drying machine, removing excess water from their surface. This step is crucial to prevent labelling issues and enhance the final presentation.
37. **X-ray detection:** the containers pass through an X-ray detection system to identify non-metallic foreign bodies that may have been missed in earlier stages, ensuring the safety of the final product.
38. **Labelling:** during the labelling stage, the corresponding labels are applied to the containers. labelling machines are adjustable according to the type of container and apply both front and back labels.
39. **Palletizing:** the labelled containers are grouped into boxes and placed on pallets using automatic palletizing systems. Robotic palletizers speed up this process and ensure efficient stacking for storage and transport.
40. **Storage:** the pallets are stored in designated storage areas at room temperature until distribution. Sometimes the product is quarantined for 1 week before distribution to ensure its stability.
41. **Shipping:** finally, the products are loaded onto trucks for distribution to retail outlets. The transport is conducted under controlled conditions to maintain product integrity during shipping.

https://doi.org/10.3390/xxxxx

S3. Description of each stage in black pitted Californian-style olive processing

Californian-style processing is similar in most stages to Spanish-style. Stages 1 to 10 are the same in both process with the difference in the stage 2 that cans are received instead of glass jars, and in stage 3 -chemicals reception- ferrous gluconate ($FeC_{12}H_{22}O_{14}$), calcium chloride: $CaCl_2$ and CO_2 are received. These stages are followed by the next two which are specific for Californian-style:

I.- Storage in fiberglass tanks (acidified brines): Olives are stored in fiberglass tanks containing acidified brine (0-1% NaCl and pH below 4.0). During this stage, pH, salt concentration, and free acidity are monitored. Controlled air blowing (0,2 l/h per litre of the tank) is applied, promoting a partial aerobic fermentation process.

II.- Oxidation and color fixation (aeration tanks): Olives are transferred to stainless steel tanks for controlled oxidation. During this process, an alkaline treatment with sodium hydroxide (NaOH) is applied while aeration is performed. The pH is then adjusted with acetic acid to around 6-7 units, and iron salts (ferrous gluconate 0.1% w/v) are added to develop the characteristic shiny black color of the final product. There is a control dosage implemented for this additive. The process consists of alternating cycles of aeration with air and CO_2, followed by resting periods in brine to ensure uniform coloration. Key parameters such as pH (neutral) and temperature are continuously monitored during oxidation. Once oxidation is finished, pH is controlled and adjusted to obtain a neutral value (6-7 units).

After oxidation, stages 17 to 34 are the same to Spanish-style processing, taking in account that this product is packed in cans instead of glass jars. After packaging and sealing, Californian-style olives are thermically treated in a sterilization process:

III.- Sterilization: The sealed containers undergo a sterilization process, where they are exposed to temperatures of 121ºC for 15 min to eliminate pathogenic microorganisms and bacterial spores, ensuring the microbiological stability of the product. This thermal treatment is more rigorous than pasteurization, allowing for an extended shelf life of the product under ambient storage conditions.

After oxidation, stages 36 to 41 are the same to Spanish-style processing.

https://doi.org/10.3390/xxxxx

Figure S1. Process flow chart for green pitted Sevillian-style olives

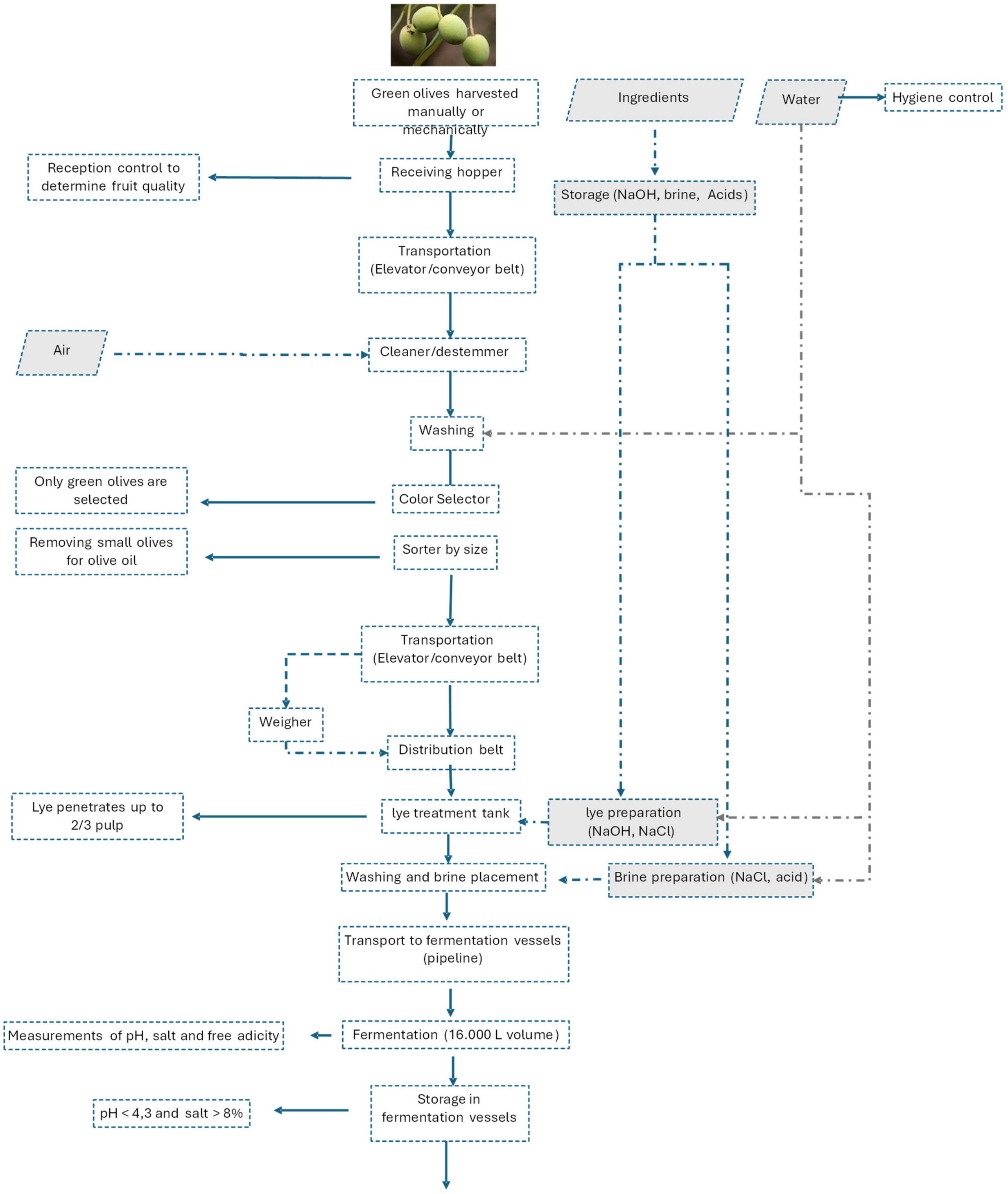

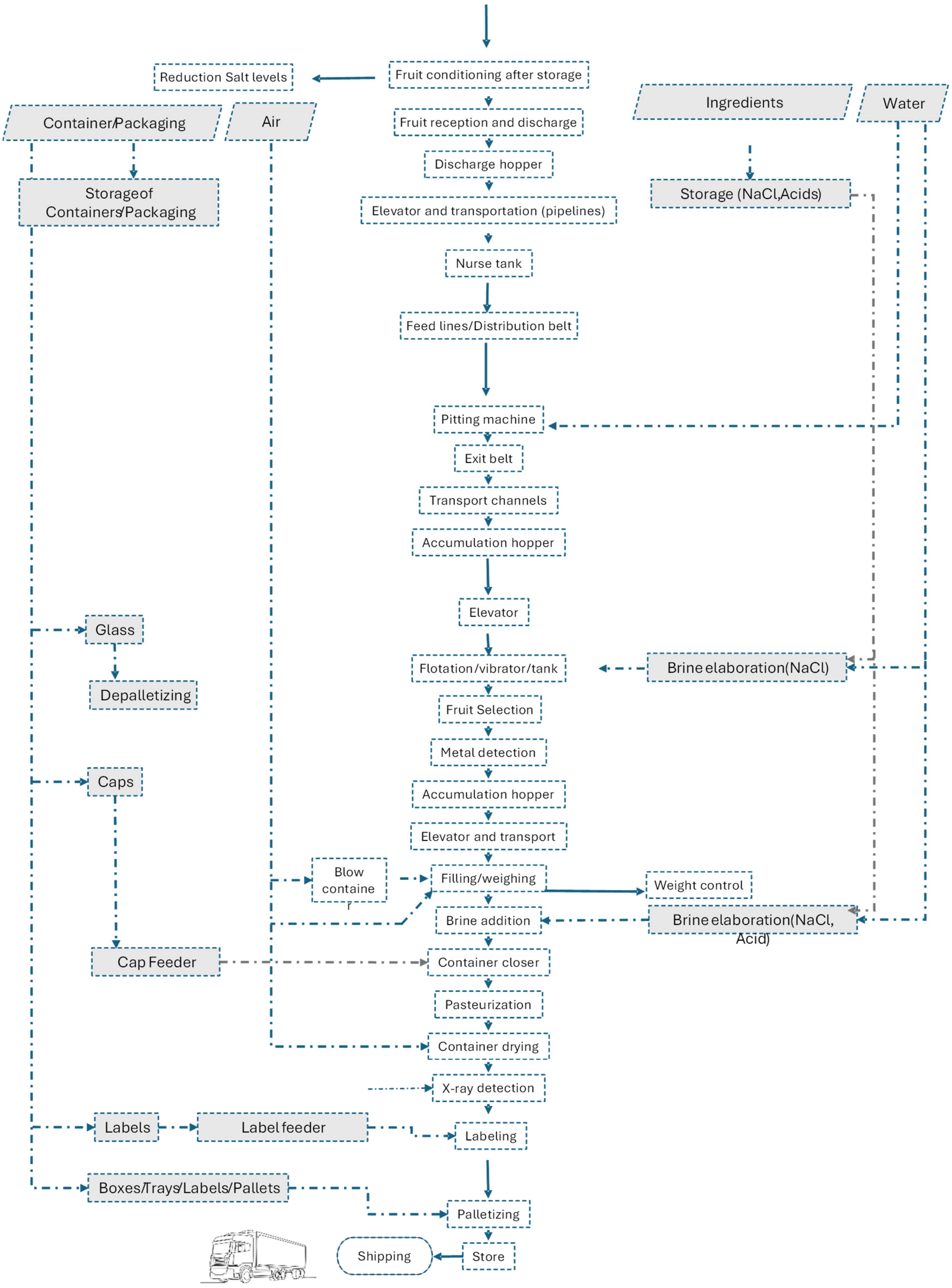
Fruit conditioning after storage
Reduction Salt levels
Container/Packaging
Air
Ingredients
Water
Fruit reception and discharge
Storage of Containers/Packaging
Discharge hopper
Storage (NaCl,Acids)
Elevator and transportation (pipelines)
Nurse tank
Feed lines/Distribution belt
Pitting machine
Exit belt
Transport channels
Accumulation hopper
Elevator
Glass
Flotation/vibrator/tank
Brine elaboration(NaCl)
Depalletizing
Fruit Selection
Metal detection
Caps
Accumulation hopper
Elevator and transport
Blow containe r
Filling/weighing
Weight control
Brine addition
Brine elaboration(NaCl, Acid)
Cap Feeder
Container closer
Pasteurization
Container drying
X-ray detection
Labels
Label feeder
Labeling
Boxes/Trays/Labels/Pallets
Palletizing
Shipping
Store

Figure S2. Process flow chart for black pitted Californian-style olives

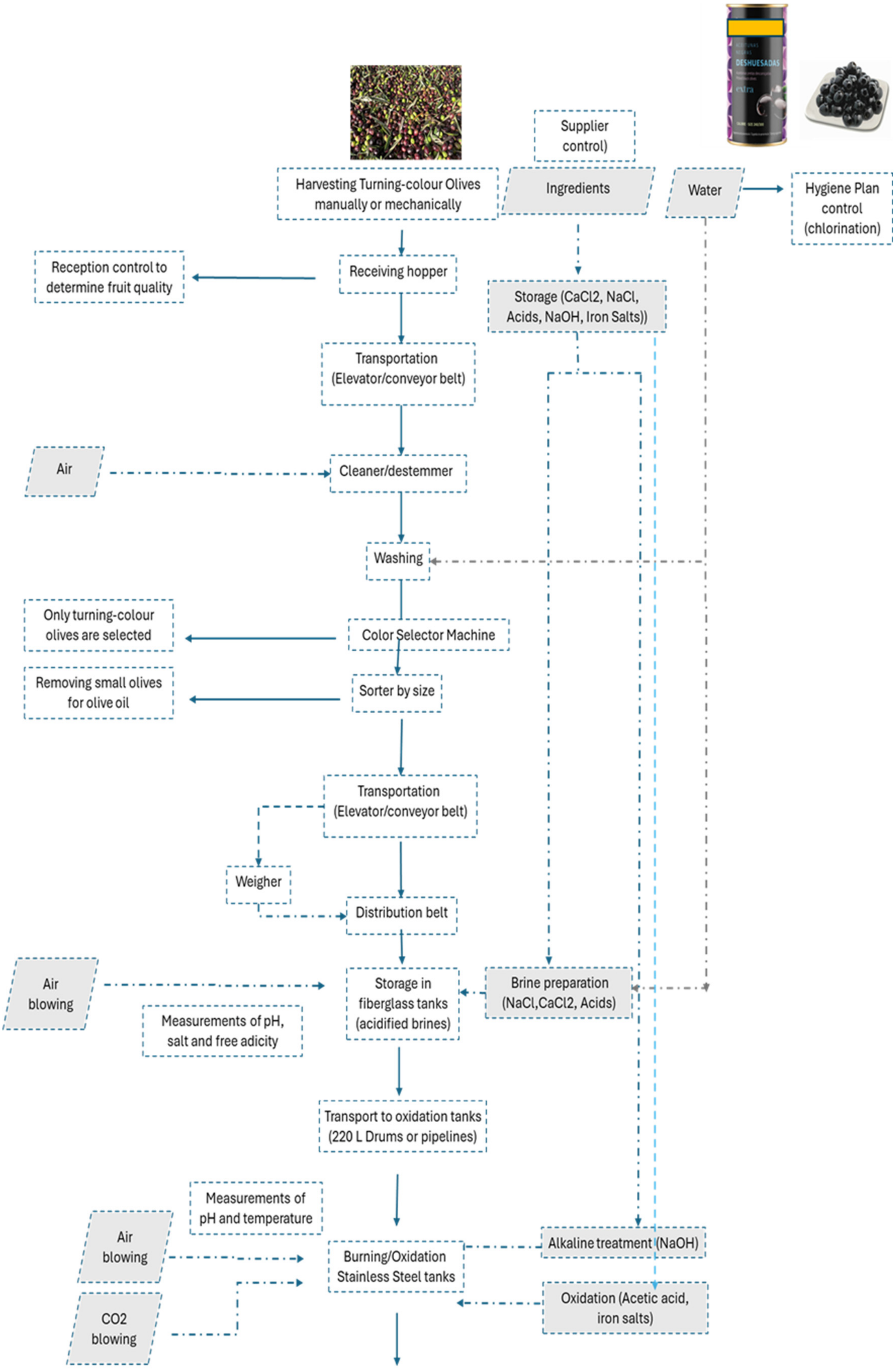

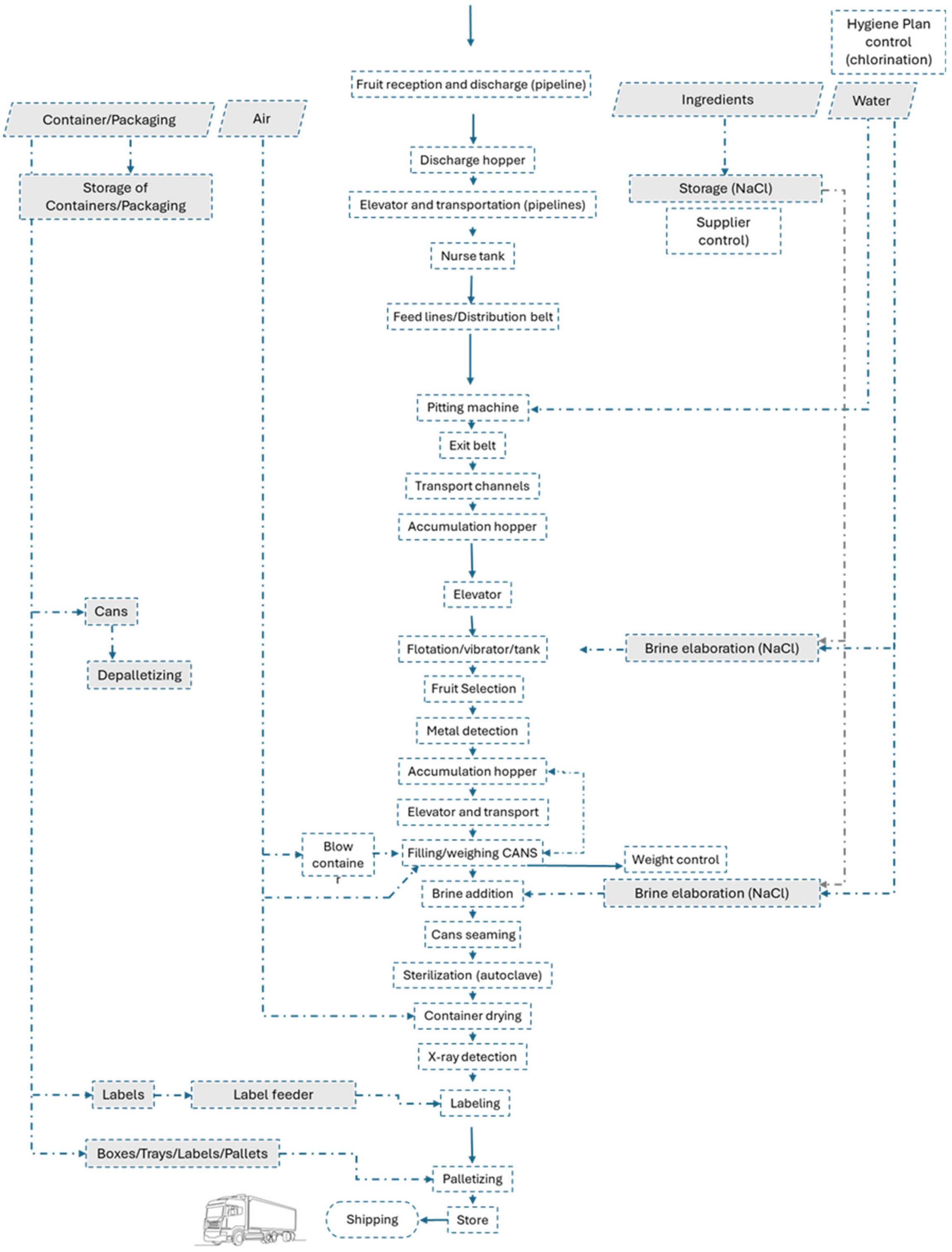
Hygiene Plan control (chlorination)
Fruit reception and discharge (pipeline)
Ingredients
Water
Container/Packaging
Air
Discharge hopper
Storage of Containers/Packaging
Elevator and transportation (pipelines)
Storage (NaCl)
Supplier control)
Nurse tank
Feed lines/Distribution belt
Pitting machine
Exit belt
Transport channels
Accumulation hopper
Elevator
Cans
Flotation/vibrator/tank
Brine elaboration (NaCl)
Depalletizing
Fruit Selection
Metal detection
Accumulation hopper
Elevator and transport
Blow containe r
Filling/weighing CANS
Weight control
Brine addition
Brine elaboration (NaCl)
Cans seaming
Sterilization (autoclave)
Container drying
X-ray detection
Labels
Label feeder
Labeling
Boxes/Trays/Labels/Pallets
Palletizing
Shipping
Store

Table S1. 4x4 risk matrix for hazards.

Immediate danger that hazard will occur (4)	4-D Medium	4-C Major	4-B Major	4-A Major
Probably will occur in time if not corrected (3)	3-D Minor	3-C Medium	3-B Major	3-A Major
Possible to occur in time if not corrected (2)	2-D Minor	2-C Medium	2-B Medium	2-A Major
Unlikely to occur; may assume" hazard will not occur (1)	1-D Minor	1-C Minor	1-B Minor	1-A Medium
**Likelihood** / **Severity**	Illness or injury is minor (D)	Illness of injury may occur, but impact is reversible (C)	Danger and illness may be severe, but it is not imminent or immediate (B)	Imminent and immediate danger of death or severe illness (A)

Table S2. Risk matrix based on FMEA.

Severity of risk		Likelihood of occurrence		Likelihood of detection	
Value	Criterion	Value	Criterion	Value	Criterion
1	None. The safety characteristics of the product are not affected.	1	Remote. There is no documentary history to show that the danger occurred before.	1	Existing control measures will almost certainly detect deviation from product safety parameters at a specific process step.
2	Mild. The safety of the final product is not affected, but there are deviations in the manufacturing procedures. It includes minor defects that lead to customer dissatisfaction	2	Unlikely. These correspond to extremely isolated incidents.	2	High probability that designs control will detect deviation from product safety parameters at a specific process stage.
3	Moderate. Product safety may potentially be compromised. More research or safety confirmation is needed before release or storage.	3	Occasional. The danger has been observed and detected before.	3	Moderate probability that designs control will detect deviation from product safety parameters at a specific process step.
4	Severe. The results of the process or product do not meet customer specifications, and the results warrant rejection of the product.	4	Common. The danger presents a certain recurrence in appearing.	4	Remote or very low probability that design control will detect deviation from safety parameters in the product at a specific stage of the process.
5	Very severe. Failure in the process potentially affects food safety. It involves legal breaches or damage to the consumer's health.	5	Frequent. Danger is inevitable and presents itself consistently.	5	Existing control measures will not fully detect deviation from product safety parameters at a specific process step.

Table S3. Hazard identification and control measures already implemented in Spanish-style olive processing

Stage	Hazards	Control Measures Already Implemented
1.- Olive receiving hopper	(P) Large debris such as branches and leaves	Separation of large debris in receiving hoppers.
	(C) Heavy metals, pesticides and mycotoxins	Supplier approval plan<br>Reception plan
	(B) Microorganisms (*Escherichia coli, Salmonella, Clostridium…*)	
2.- Reception of packaging and labelling materials	(P) Foreign bodies	Supplier approval plan<br>Reception control plan
	(C) Chemical migration	
	(B) Microorganisms (bacteria, virus, molds and yeasts) and pests	
3.- Sodium hydroxide (NaOH), chemicals and salt (NaCl) reception	(P) None	None
	(C) Incorrect chemical	Supplier approval plan<br>Reception control plan
	(B) None	None
4.- Transportation elevator/conveyor belt<br>5.-Cleaner/destemmer	(P) Foreign bodies and small debris such as leaves and branches	Preventative maintenance
	(C) Equipment chemicals and remains of cleaning and disinfection products	Preventative maintenance<br>Cleaning and disinfection plan
	(B) Microorganisms	Cleaning and disinfection plan
6.- Washing (I)	(P) Foreign bodies	Preventative maintenance
	(C) Heavy metals	Water control plan
	(B) Microorganisms (*Escherichia coli, Clostridium perfringens…*)	
7.- Sorter<br>8.- Colour selector<br>9.- Transportation (elevator/conveyor belt)	(P) Foreign bodies	Preventative maintenance
	(C) Equipment chemicals and remains of cleaning and disinfection products	Preventative maintenance<br>Cleaning and disinfection plan
	(B) Microorganisms (mainly mesophilic aerobes)	Cleaning and disinfection plan
10.-Lye treatment	(P) Foreign bodies	Preventative maintenance

	(C) Equipment chemicals and remains of cleaning and disinfection products	Preventative maintenance<br><br>Cleaning and disinfection plan
	(B) None	None
11.- Washing (II)	(P) Foreign bodies	Preventative maintenance
	(C) Heavy metals	Water control plan
	(B) Microorganisms (*Escherichia coli, Clostridium perfringens*…)	Water control plan
12.- Brine placement	(P) Foreign bodies	Preventative maintenance
	(Q) None	None
	(B) Spoilage microorganisms (*Enterobacteriaceae, Celerinatantimonas,* etc.).	Dosage control of NaCl (8-10%) and HCl addition.
13.- Transport to fermentation vessels (Pipeline)	(P) Foreign bodies	Preventative maintenance
	(C) Equipment chemicals and remains of cleaning and disinfection products	Preventative maintenance<br><br>Cleaning and disinfection plan
	(B) Microorganisms (mainly mesophilic aerobes)	Cleaning and disinfection plan
14.- Fermentation	(P) Foreign bodies	Preventative maintenance
	(Q) Remains of cleaning and disinfection products	Cleaning and disinfection plan
	(B) Microorganisms<br><br>(*Enterobacteriaceae, Clostridium, Pseudomonas, Staphylococcus,* etc.)	Control of pH, salt and free and combined acidity.<br><br>Control of absence of bad odors and flavors.<br><br>Removal of fermenting surface veils
15.- Storage in fermentation vessels	(P) Foreign bodies	Preventative maintenance
	(Q) Remains of cleaning and disinfection products	Cleaning and disinfection plan
	(B) Microorganisms (*Propionibacterias, aerobic fungi, Clostridium*)	Control of pH (<4,3) and NaCl (>8%)<br><br>Removal of fermenting surface veils

16.- Fruit conditioning after storage	(P) Foreign bodies	Preventative maintenance
	(Q) Equipment chemicals and remains of cleaning and disinfection products	Preventative maintenance<br>Cleaning and disinfection plan
	(B) Microorganisms (*Clostridium, Staphylococcus, Pseudomonas, Enterobacteriaceae*)	Control of pH (<4,3) and NaCl (4-5%).<br>Absence of fermentable sugars.
17.- Fruit reception and discharge	(P) Foreign bodies	Preventative maintenance
	(Q) Remains of cleaning and disinfection products	Cleaning and disinfection plan
	(B) Microorganisms (*Clostridium, Staphylococcus, Pseudomonas, Enterobacteriaceae*)	Control of pH and NaCl<br>Cleaning and disinfection plan
18.- Discharge hopper<br>19.- Elevator and transportation (pipelines)<br>20.- Nurse tank<br>21.- Feed lines/distribution belt<br>22.- Pitting machine<br>23.- Exit belt	(P) Foreign bodies	Preventative maintenance
	(C) Equipment chemicals and remains of cleaning and disinfection products	Preventative maintenance<br>Cleaning and disinfection plan
	(B) Microorganisms (mainly mesophilic aerobes)	Cleaning and disinfection plan
24.- Transport channels	(P) Foreign bodies	Preventative maintenance
	(C) Equipment chemicals and remains of cleaning and disinfection products<br>Heavy metals of water	Preventative maintenance<br>Cleaning and disinfection plan<br>Water control plan
	(B) Microorganisms	Cleaning and disinfection plan<br>Water control plan
25.- Accumulation hopper<br>26.- Elevator	(P) Foreign bodies	Preventative maintenance
	(C) Equipment chemicals and remains of cleaning and disinfection products	Preventative maintenance<br>Cleaning and disinfection plan
	(B) Microorganisms (mainly mesophilic aerobes)	Cleaning and disinfection plan

27.- Flotation/vibrator tank	(P) Foreign bodies	Preventative maintenance
	(C) Equipment chemicals and remains of cleaning and disinfection products<br>Heavy metals of water	Preventative maintenance<br>Cleaning and disinfection plan<br>Water control plan
	(B) Microorganisms	Cleaning and disinfection plan<br>Water control plan
28.- Fruit selection	(P) Foreign bodies	Preventative maintenance
	(C) Equipment chemicals and remains of cleaning and disinfection products	Preventative maintenance<br>Cleaning and disinfection plan
	(B) Microorganisms (mainly mesophilic aerobes)	Cleaning and disinfection plan
29.- Metal detection	(P) Foreign metal bodies	Metal detection
	(C) Remains of cleaning and disinfection products	Cleaning and disinfection plan
	(B) Microorganisms (mainly mesophilic aerobes)	Cleaning and disinfection plan
30.- Accumulation hopper<br>31.- Elevator and transport<br>32.- Filling/weighing	(P) Foreign bodies	Preventative maintenance
	(C) Equipment chemicals and remains of cleaning and disinfection products	Preventative maintenance<br>Cleaning and disinfection plan
	(B) Microorganisms (mainly mesophilic aerobes)	Cleaning and disinfection plan
33.- Brine addition	(P) Foreign bodies	Preventative maintenance
	(C) Equipment chemicals and remains of cleaning and disinfection products	Preventative maintenance<br>Cleaning and disinfection plan
	(B) Microorganisms (mainly mesophilic aerobes)	Cleaning and disinfection plan<br>Control of the brine [Nacl (4-5%), pH (<4,3) and acidity (0,3-0,5%)].
34.-Container sealing	(P) Foreign bodies	Preventative maintenance
	(C) Equipment chemicals and remains of cleaning and disinfection products	Preventative maintenance<br>Cleaning and disinfection plan
	(B) Microorganisms	Control of hermetic sealing

35.- Pasteurization	(P) None	None
	(C) None	None
	(B) Microorganisms (*Clostridium,* aerobic mesophiles, fungal spores)	Temperature: 70-85ºC<br>Time: 5-20 minutes<br>15 PU units
36.- Container drying	(P) Glass	Preventative maintenance<br>Good manufacturing practices
	(C) None	None
	(B) None	None
37.- X-ray detection	(P) Foreign bodies	X-ray detection
	(C) None	None
	(B) None	None
38.-Labelling<br>39.- Palletizing<br>40.- Storage<br>41.- Shipping	(P) Glass	Good manufacturing practices
	(C) None	None
	(B) None	None

https://doi.org/10.3390/xxxxx

Table S4. Hazard analysis after applying 4x4 matrix and FMEA model in Spanish-style olive processing

Stage	Hazard	Control measures	4x4 Matrix model				FMEA model				
			Probability	Severity	Risk	SIG	Probability	Severity	Detection	CI	SIG
1.- Olive receiving hopper	(P) Large debris such as branches and leaves	Separation of large debris in receiving hoppers	3 Probably. The danger presents a certain recurrence in appearing as described [41, 42]	D Minor. The safety of the final product is not affected because these foreign bodies are usually of considerable size and are detected by consumers. .	3D Minor	No	4 Common. The danger presents a certain recurrence in appearing as described [41, 42]	Mild 2. The safety of the final product is not affected because these foreign bodies are usually of considerable size and are detected by consumers.	1 Existing detection measures. At the end of the process there is an X-ray machine for the detection of foreign bodies.	8	No
	(C) Heavy metals, pesticides and mycotoxins	Supplier aproval plan<br>Reception plan	3 Probably. The danger has been observed and detected before as described [45, 46, 48, 52]	B Severe not imminent as described [40, 45, 47, 48, 49]	3B<br>Major	Yes	3 Occasional. The danger has been observed and detected before as described [45, 46, 48, 52]	5 Very severe. Failure in the process potentially affects food safety. It involves legal breaches or	1 Existing detection measures. The supplier control plan includes analysis to detect these hazards in olives.	15	Yes<br>S=5

Stage	Hazard	Control measures	4x4 Matrix model				FMEA model				
			**Probability**	**Severity**	**Risk**	**SIG**	**Probability**	**Severity**	**Detection**	**CI**	**SIG**
								damage to the consumer's health as described [40, 45, 47, 48, 49]			
	(B) Microorganisms (*Escherichia coli, Salmonella, Clostridium…*)	Supplier aproval plan<br><br>Reception plan	2 Possible to occur in time if not corrected [61]	A Severe. Imminent and immediate danger of death or severe illness as described [40]	2A<br><br>Major	Yes	3 Occasional. The danger has been observed and detected before as described [61]	5 Very severe. Failure in the process potentially affects food safety. It involves legal breaches or damage to the consumer's health as described [40]	1 Existing detection measures. The supplier control plan includes analysis to detect these hazards in olives.	15	Yes<br><br>S=5
2.- Reception of packaging and labelling materials	(P) Foreign bodies	Supplier approval plan<br><br>Reception control plan	2 Possible to occur in time if not corrected [44]	A Severe. Glass fragments can cause significant injuries to the	2A Major	Yes	3 Occasional. The danger has been observed and detected be-	5 Very severe. Glass fragments can cause significant injuries to	1 Existing detection measures. The supplier approval plan and	15	Yes<br><br>S=5

Stage	Hazard	Control measures	4x4 Matrix model				FMEA model				
			Probability	Severity	Risk	SIG	Probability	Severity	Detection	CI	SIG
				oral cavity and gastro-intestinal tract.			fore as described [44]	the oral cavity and gastrointestinal tract.	reception control plan include the control of these hazards.		
	(C) Chemical migration	Supplier approval plan<br><br>Reception control plan	2 Possible to occur in time if not corrected [55]	B Severe not imminent as described [55, 56]	2B Medium	Yes	2 Unlikely. These correspond to extremely isolated incidents [55]	5 Very severe. Failure in the process potentially affects food safety. It involves legal breaches or damage to the consumer's health as described [55, 56]	1 Existing detection measures.<br>The supplier approval plan includes the control of these hazards.	10	Yes<br><br>S=5
	(B) Microoganisms (bacteria, virus, molds and yests) and pests	Supplier approval plan<br><br>Reception control plan	1 Unlikely to occur; may assume" hazard will not occur	C Reversible	1C<br><br>Minor	No	2 Unlikely to occur; may assume" hazard will not occur	3 Moderate. Product safety may potentially be compromised.	1 Existing detection measures.<br>The supplier approval plan includes the control of	6	No

Stage	Hazard	Control measures	4x4 Matrix model				FMEA model				
			Probability	Severity	Risk	SIG	Probability	Severity	Detection	CI	SIG
									these hazards.		
3.- Reception of sodium hydroxide (NaOH) and salt (NaCl) reception	(C) Incorrect chemical	Supplier approval plan<br>Reception control plan	1 Unlikely to occur; may assume" hazard will not occur	A Severe. Imminent and immediate danger of death or severe illness.	1A Medium	Yes	2 Unlikely. These correspond to extremely isolated incidents.	5 Very severe. Failure in the process potentially affects food safety. It involves legal breaches or damage to the consumer's health	1 Existing detection measures. The supplier approval plan includes the control of these hazards.	10	Yes<br>S=5
4.- Transportation elevator/conveyor belt	(P) Foreign bodies	Preventative maintenance	2 Possible to occur in time if not corrected [44]	A Severe. Foreign bodies can cause significant injuries to the oral cavity and gastrointestinal tract.	2A Major	Yes	3 Occasional. The danger has been observed and detected before as described [44]	5 Very severe. Foreign bodies can cause significant injuries to the oral cavity and gastrointestinal tract.	1 Existing detection measures. At the end of the process there is a metal detector and an X-ray machine for the detection of	15	Yes<br>S=5

Stage	Hazard	Control measures	4x4 Matrix model				FMEA model				
			Probability	Severity	Risk	SIG	Probability	Severity	Detection	CI	SIG
									any foreign bodies		
	(C) Equipment chemicals and remains of cleaning and disinfection products	Preventative maintenance<br><br>Cleaning and disinfection plan<br><br>Training plan	1 Unlikely to occur; may assume hazard will not occur due to the correct implementation of the PPRs for maintenance, cleaning and disinfection and staff training [58]	D Minor. The potential contamination would be residual and products used are authorized [57].	1D Minor	No	2 Unlikely. These correspond to extremely isolated incidents due to the correct implementation of the PPRs for maintenance, cleaning and disinfection and staff training [58]	2 Mild. The potential contamination would be residual and products used are authorized [57].	5 There are no detection measures	20	No
	(B) Microorganisms	Cleaning and disinfection plan<br><br>Training plan	1 Unlikely to occur; may assume hazard will not occur due to the correct implementation of the PPRs for cleaning and	D Minor. The potential contamination would be residual.	1D Minor	No	2 Unlikely. These correspond to extremely isolated incidents due to the correct implementation of the PPRs for	2 Mild. The potential contamination would be residual.	5 There are no detection measures	20	No

Stage	Hazard	Control measures	4x4 Matrix model				FMEA model				
			Probability	Severity	Risk	SIG	Probability	Severity	Detection	CI	SIG
			disinfection and staff training.				cleaning and disinfection and staff training.				
5.- Cleaner/ destemmer	(P) Foreign bodies and small debris such as leaves and branches	Preventative maintenance<br>Vibrating rollers	2 Possible to occur in time if not corrected [41, 42]	A Severe. Foreign bodies can cause significant injuries to the oral cavity and gastro-intestinal tract.	2A Major	Yes	3 Occasional. The danger has been observed and detected before as described [41,42]	5 Very severe. Foreign bodies can cause significant injuries to the oral cavity and gastrointestinal tract.	1 Existing detection measures. At the end of the process there is a metal detector and an X-ray machine for the detection of any foreign bodies	15	Yes<br>S=5
	(C) Equipment chemicals and remains of cleaning and disinfection products	Preventative maintenance<br>Cleaning and disinfection plan<br>Training plan	1 Unlikely to occur; may assume hazard will not occur due to the correct implementation of the PPRs for maintenance, cleaning and disinfection	D Minor. The potential contamination would be residual and products used are authorized [57].	1D Minor	No	2 Unlikely These correspond to extremely isolated incidents due to the correct implementation of the PPRs for maintenance, cleaning and	2 Mild. The potential contamination would be residual and products used are authorized [57].	5 There are no detection measures	20	No

Stage	Hazard	Control measures	4x4 Matrix model				FMEA model				
			Probability	Severity	Risk	SIG	Probability	Severity	Detection	CI	SIG
			and staff training [58]				disinfection and staff training [58]				
	(B) Microorganisms	Cleaning and disinfection plan<br><br>Training plan	1 Unlikely to occur; may assume hazard will not occur due to the correct implementation of the PPRs for cleaning and disinfection and staff training.	D Minor. The potential contamination would be residual.	1D Minor	No	2 Unlikely These correspond to extremely isolated incidents due to the correct implementation of the PPRs for cleaning and disinfection.	2 Mild. The potential contamination would be residual.	5 There are no detection measures	20	No
6.- Washing (I)	(P) Foreign bodies	Preventative maintenance	2 Possible to occur in time if not corrected [44]	A Severe. Foreign bodies can cause significant injuries to the oral cavity and gastro-intestinal tract.	2A Major	Yes	3 Occasional. The danger has been observed and detected before as described [44]	5 Very severe. Foreign bodies can cause significant injuries to the oral cavity and gastrointestinal tract.	1 Existing detection measures. At the end of the process there is a metal detector and an X-ray machine for the detection of	15	Yes<br><br>S=5

Stage	Hazard	Control measures	4x4 Matrix model				FMEA model				
			Probability	Severity	Risk	SIG	Probability	Severity	Detection	CI	SIG
									any foreign bodies		
	(C) Heavy metals	Water control plan	2 Possible to occur in time if not corrected [50]	B Severe not imminent as described [40, 45, 47, 48, 49]	2B Medium	Yes	3 Occasional The danger has been observed and detected before as described [50]	5 Very severe. Failure in the process potentially affects food safety. It involves legal breaches or damage to the consumer's health as described [40, 45, 47, 48, 49]	1 Existing detection measures. Water control plan includes analysis to determine heavy metals	15	Yes S=5
	(B) Microoganisms (*Escherichia coli, Clostridium perfringens…*)	Water control plan	2 Possible to occur in time if not corrected [40]	A Severe. Imminent and immediate danger of death or severe illness as	2A Major	Yes	3 Occasional. The danger has been observed and detected before [40].	5 Very severe. Failure in the process potentially affects food safety. It involves legal	1 Existing detection measures. Water control plan includes analysis to determine	15	Yes S=5

Stage	Hazard	Control measures	4x4 Matrix model				FMEA model				
			Probability	Severity	Risk	SIG	Probability	Severity	Detection	CI	SIG
				described [40]				breaches or damage to the consumer's health as described [40]	food-born pathogens.		
7.- Sorter	(P) Foreign bodies	Preventative maintenance	2 Possible to occur in time if not corrected [44]	A Severe. Foreign bodies can cause significant injuries to the oral cavity and gastro-intestinal tract.	2A Major	Yes	3 Occasional. The danger has been observed and detected before as described [44]	5 Very severe. Foreign bodies can cause significant injuries to the oral cavity and gastrointestinal tract.	1 Existing detection measures. At the end of the process there is a metal detector and an X-ray machine for the detection of any foreign bodies	15	Yes S=5
	(C) Equipment chemicals and remains of cleaning and disinfection products	Preventative maintenance Cleaning and disinfection plan Training plan	1 Unlikely - to occur; may assume hazard will not occur due to the correct implementation of the PPRs for	D Minor. The potential contamination would be residual and products used	1D Minor	No	2 Unlikely These correspond to extremely isolated incidents due to the correct implementation of the	2 Mild. The potential contamination would be residual and products used are authorized [57].	5 There are no detection measures	20	No

Stage	Hazard	Control measures	4x4 Matrix model				FMEA model				
			Probability	Severity	Risk	SIG	Probability	Severity	Detection	CI	SIG
			maintenance cleaning and disinfection and staff training [58]	are authorized [57].			PPRs for maintenance, cleaning and disinfection and staff training [58]				
	(B) Microorganisms (mainly mesophilic aerobes)	Cleaning and disinfection plan<br>Training plan	1 Unlikely to occur; may assume hazard will not occur due to the correct implementation of the PPRs for cleaning and disinfection and staff training.	D Minor. The potential contamination would be residual.	1D Minor	No	2 Unlikely. These correspond to extremely isolated incidents due to the correct implementation of the PPRs for cleaning and disinfection and staff training.	2 Mild. The potential contamination would be residual.	5 There are no detection measures	20	No
8.- Colour selector	(P) Foreign bodies	Preventative maintenance	2 Possible to occur in time if not corrected [44]	A Severe. Foreign bodies can cause significant injuries to the oral cavity and	2A Major	Yes	3 Occasional. The danger has been observed and detected before as described [44]	5 Very severe. Foreign bodies can cause significant injuries to the oral cavity and	1 Existing detection measures. At the end of the process there is a metal detector and an X-ray	15	Yes<br>S=5

Stage	Hazard	Control measures	4x4 Matrix model				FMEA model				
			Probability	Severity	Risk	SIG	Probability	Severity	Detection	CI	SIG
				gastrointestinal tract.				gastrointestinal tract.	machine for the detection of any foreign bodies		
	(C) Equipment chemicals and remains of cleaning and disinfection products	Preventative maintenance<br><br>Cleaning and disinfection plan<br><br>Training plan	1 Unlikely - to occur; may assume hazard will not occur due to the correct implementation of the PPRs for maintenance, cleaning and disinfection and staff training [58]	D Minor. The potential contamination would be residual and products used are authorized [57].	1D Minor	No	2 Unlikely These correspond to extremely isolated incidents due to the correct implementation of the PPRs for maintenance, cleaning and disinfection and staff training [58]	2 Mild. The potential contamination would be residual and products used are authorized [57].	5 There are no detection measures	20	No
	(B) Microorganisms (mainly mesophilic aerobes)	Cleaning and disinfection plan<br><br>Training plan	1 Unlikely to occur; may assume hazard will not occur due to the correct implementation of the PPRs for	D Minor. The potential contamination would be residual.	1D Minor	No	2 Unlikely. These correspond to extremely isolated incidents due to the correct implementation of the	2 Mild. The potential contamination would be residual.	5 There are no detection measures	20	No

Stage	Hazard	Control measures	4x4 Matrix model				FMEA model				
			Probability	Severity	Risk	SIG	Probability	Severity	Detection	CI	SIG
			cleaning and disinfection and staff training.				PPRs for cleaning and disinfection and staff training.				
9.-Transportation (elevator/conveyor belt)	(P) Foreign bodies	Preventative maintenance	2 Possible to occur in time if not corrected [44]	A Severe. Foreign bodies can cause significant injuries to the oral cavity and gastrointestinal tract.	2A Major	Yes	3 Occasional. The danger has been observed and detected before as described [44]	5 Very severe. Foreign bodies can cause significant injuries to the oral cavity and gastrointestinal tract.	1 Existing detection measures. At the end of the process there is a metal detector and an X-ray machine for the detection of any foreign bodies	15	Yes S=5
	(C) Equipment chemicals and remains of cleaning and disinfection products	Preventative maintenance<br>Cleaning and disinfection plan<br>Training plan	1 Unlikely - to occur; may assume hazard will not occur due to the correct implementation of the PPRs for for maintenance,	D Minor. The potential contamination would be residual and products used are authorized [57]. Failure in	1D Minor	No	2 Unlikely These correspond to extremely isolated incidents due to the correct implementation of the PPRs for maintenance,	2 Mild. The potential contamination would be residual and products used are authorized [57].	5 There are no detection measures	20	No

Stage	Hazard	Control measures	4x4 Matrix model				FMEA model				
			Probability	Severity	Risk	SIG	Probability	Severity	Detection	CI	SIG
			cleaning and disinfection and staff training [58]	the process po-tentially affects food safety. It in-volves legal breaches or damage to the con-su-mer's health			cleaning and disinfection and staff training [58]				
	(B) Microorganisms (mainly mesophilic aerobes)	Cleaning and disinfection plan<br><br>Training plan	1 Unlikely to occur; may assume haz-ard will not occur due to the correct implementa-tion of the PPRs for cleaning and disinfection and staff training.	D Minor. The poten-tial contam-ination would be residual.	1D Minor	No	2 Unlikely. These corre-spond to ex-tremely iso-lated inci-dents due to the correct implementa-tion of the PPRs for cleaning and disinfection and staff training.	2 Mild. The potential contamina-tion would be residual.	5 There are no detection measures	20	No
10.- Lye treatment	(P) Foreign bodies	Preventative maintenance	2 Possible to occur in time if not cor-rected [44]	A Severe. Foreign bodies can cause	2A Major	Yes	3 Occasional. The danger has been ob-served and	5 Very se-vere. For-eign bodies can cause	1 Existing de-tection measures. At the end of the	15	Yes<br><br>S=5

**Stage**	**Hazard**	**Control measures**	**4x4 Matrix model**				**FMEA model**				
			**Probability**	**Severity**	**Risk**	**SIG**	**Probability**	**Severity**	**Detection**	**CI**	**SIG**
				significant injuries to the oral cavity and gastrointestinal tract.			detected before as described [44]	significant injuries to the oral cavity and gastrointestinal tract.	process there is a metal detector and an X-ray machine for the detection of any foreign bodies		
	(C) Equipment chemicals and remains of cleaning and disinfection products	Preventative maintenance<br><br>Cleaning and disinfection plan<br><br>Training plan	1 Unlikely - to occur; may assume hazard will not occur due to the correct implementation of the PPRs for for maintenance, cleaning and disinfection and staff training [58]	D Minor. The potential contamination would be residual and products used are authorized [57].	1D Minor	No	2 Unlikely These correspond to extremely isolated incidents due to the correct implementation of the PPRs for maintenance, cleaning and disinfection and staff training [58]	2 Mild. The potential contamination would be residual and products used are authorized [57].	5 There are no detection measures	20	No
11.- Washing (II)	(P) Foreign bodies	Preventative maintenance	2 Possible to occur in time if not corrected [44]	A Severe. Foreign bodies can cause significant	2A Major	Yes	3 Occasional. The danger has been observed and detected be-	5 Very severe. Foreign bodies can cause significant	1 Existing detection measures. At the end of the process there	15	Yes<br><br>S=5

Stage	Hazard	Control measures	4x4 Matrix model				FMEA model				
			Probability	Severity	Risk	SIG	Probability	Severity	Detection	CI	SIG
				injuries to the oral cavity and gastrointestinal tract.			fore as described [44]	injuries to the oral cavity and gastrointestinal tract.	is a metal detector and an X-ray machine for the detection of any foreign bodies		
	(C) Heavy metals	Water control plan	2 Possible. to occur in time if not corrected [50]	B Severe not imminent as described [40, 45, 47, 48, 49]	2B Medium	Yes	3 Occasional The danger has been observed and detected before as described [50]	5 Very severe. Failure in the process potentially affects food safety. It involves legal breaches or damage to the consumer's health as described [40, 45, 47, 48, 49]	1 Existing detection measures. Water control plan includes analysis to determine these hazards.	15	Yes S=5
	(B) Microorganisms (*Escherichia coli, Clostridium perfringens*…)	Water control plan	2 Possible to occur in time if not corrected [40]	A Severe. Imminent and immediate danger of death	2A Major	Yes	3 Occasional. The danger has been observed and	5 Very severe. Failure in the process potentially	1 Existing detection measures. Water control plan includes	15	Yes S=5

Stage	Hazard	Control measures	4x4 Matrix model				FMEA model				
			Probability	Severity	Risk	SIG	Probability	Severity	Detection	CI	SIG
				or severe illness as described [40]			detected before [40].	affects food safety. It involves legal breaches or damage to the consumer's health as described [40]	analysis to determine food-born pathogens.		
12.- Brine placement	(P) Foreign bodies	Preventative maintenance	2 Possible to occur in time if not corrected [44]	A Severe. Foreign bodies can cause significant injuries to the oral cavity and gastrointestinal tract.	2A Major	Yes	3 Occasional. The danger has been observed and detected before as described [44]	5 Very severe. Foreign bodies can cause significant injuries to the oral cavity and gastrointestinal tract.	1 Existing detection measures. At the end of the process there is a metal detector and an X-ray machine for the detection of any foreign bodies	15	Yes S=5
	(B) Microorganisms	Dosage control of NaCl (8-10%)	2 Possible to occur in time if not corrected [62]	D Minor. The potential contamination would be residual.	2D Minor	No	3 Occasional. The danger has been observed and detected be-	2 Mild. The potential contamination would be residual	1 Existing detection measures. Dosage control.	6	No

Stage	Hazard	Control measures	4x4 Matrix model				FMEA model				
			Probability	Severity	Risk	SIG	Probability	Severity	Detection	CI	SIG
							fore as described [62]				
13.- Transport to fermentation vessels (Pipeline)	(P) Foreign bodies	Preventative maintenance	2 Possible to occur in time if not corrected [44]	A Severe. Foreign bodies can cause significant injuries to the oral cavity and gastro-intestinal tract.	2A Major	Yes	3 Occasional. The danger has been observed and detected before as described [44]	5 Very severe. Foreign bodies can cause significant injuries to the oral cavity and gastrointestinal tract.	1 Existing detection measures. At the end of the process there is a metal detector and an X-ray machine for the detection of any foreign bodies	15	Yes<br>S=5
	(C) Equipment chemicals and remains of cleaning and disinfection products	Preventative maintenance<br>Cleaning and disinfection plan<br>Training plan	1 Unlikely to occur; may assume hazard will not occur due to the correct implementation of the PPRs for for maintenance, cleaning and disinfection and staff training [58]	D Minor. The potential contamination would be residual and products used are authorized [57].	1D Minor	No	2 Unlikely These correspond to extremely isolated incidents due to the correct implementation of the PPRs for maintenance, cleaning and disinfection	2 Mild. The potential contamination would be residual and products used are authorized [57].	5 There are no detection measures	20	No

Stage	Hazard	Control measures	4x4 Matrix model				FMEA model				
			Probability	Severity	Risk	SIG	Probability	Severity	Detection	CI	SIG
							and staff training [58]				
	(B) Microorganisms (mainly mesophilic aerobes)	Cleaning and disinfection plan<br>Training plan	1 Unlikely to occur; may assume hazard will not occur due to the correct implementation of the PPRs for cleaning and disinfection and staff training.	D Minor. The potential contamination would be residual.	1D Minor	No	2 Unlikely. These correspond to extremely isolated incidents due to the correct implementation of the PPRs for cleaning and disinfection and staff training.	2 Mild. The potential contamination would be residual.	5 There are no detection measures	20	No
14.- Fermentation	(P) Foreign bodies	Preventative maintenance	2 Possible to occur in time if not corrected [44]	A Severe. Foreign bodies can cause significant injuries to the oral cavity and gastrointestinal tract.	2A Major	Yes	3 Occasional. The danger has been observed and detected before as described [44]	5 Very severe. Foreign bodies can cause significant injuries to the oral cavity and gastrointestinal tract.	1 Existing detection measures. At the end of the process there is a metal detector and an X-ray machine for the detection of	15	Yes<br>S=5

Stage	Hazard	Control measures	4x4 Matrix model				FMEA model				
			Probability	Severity	Risk	SIG	Probability	Severity	Detection	CI	SIG
									any foreign bodies		
	(Q) Remains of cleaning and disinfection products	Cleaning and disinfection plan<br>Training plan	1 Unlikely - to occur; may assume hazard will not occur due to the correct implementation of the PPRs for cleaning and disinfection and staff training [58]	D Minor. The potential contamination would be residual and products used are authorized [57].	1D Minor	No	2 Unlikely These correspond to extremely isolated incidents due to the correct implementation of the PPRs for cleaning and disinfection and staff training [58]	2 Mild. The potential contamination would be residual and products used are authorized [57].	5 There are no detection measures	20	No
	(B) Microorganisms<br>(*Enterobacteriaceae, Clostridium, Pseudomonas, Staphylococcus*, etc.)	Control of pH, salt and free and combined acidity.<br>Control of absence of bad odors and flavors.	2 Possible to occur in time if not corrected [64]	A Severe Imminent and immediate danger of death or severe illness as described [40]	2A Major	Yes	3 Occasional. The danger has been observed and detected before as described [64]	5 Very severe. Failure in the process potentially affects food safety. It involves legal breaches or damage to the	1 Existing detection measures.<br>pH, salt, free and combined acidity are monitored.	15	Yes<br>S=5

Stage	Hazard	Control measures	4x4 Matrix model				FMEA model				
			Probability	Severity	Risk	SIG	Probability	Severity	Detection	CI	SIG
		Removal of fermenting surface veils						consumer's health as described [40]			
15.- Storage in fermentation vessels	(P) Foreign bodies	Preventative maintenance	2 Possible to occur in time if not corrected [44]	A Severe. Foreign bodies can cause significant injuries to the oral cavity and gastro-intestinal tract.	2A Major	Yes	3 Occasional. The danger has been observed and detected before as described [44]	5 Very severe. Foreign bodies can cause significant injuries to the oral cavity and gastrointestinal tract.	1 Existing detection measures. At the end of the process there is a metal detector and an X-ray machine for the detection of any foreign bodies	15	Yes<br>S=5
	(Q) Remains of cleaning and disinfection products	Cleaning and disinfection plan<br>Training plan	1 Unlikely -to occur; may assume hazard will not occur due to the correct implementation of the PPRs for cleaning and disinfection	D Minor. The potential contamination would be residual and products used are authorized [57].	1D Minor	No	2 Unlikely These correspond to extremely isolated incidents due to the correct implementation of the PPRs for cleaning and disinfection	2 Mild. The potential contamination would be residual and products used are authorized [57].	5 There are no detection measures	20	No

Stage	Hazard	Control measures	4x4 Matrix model				FMEA model				
			Probability	Severity	Risk	SIG	Probability	Severity	Detection	CI	SIG
			and staff training [58]				and staff training [58]				
	(B) Microorganisms (*Propionibacterias, aerobic fungi, Clostridium*)	Control of pH (<4,3) and NaCl (>8%)<br>Removal of fermenting surface veils	2 Possible to occur in time if not corrected [64]	A Severe Imminent and immediate danger of death or severe illness as described [40]	2A Major	Yes	3 Occasional. The danger has been observed and detected before as described [64]	5 Very severe. Failure in the process potentially affects food safety. It involves legal breaches or damage to the consumer's health as described [40]	1 Existing detection measures. pH and salt are monitored.	15	Yes<br>S=5
16.- Fruit conditioning after storage	(P) Foreign bodies	Preventative maintenance	2 Possible to occur in time if not corrected [44]	A Severe. Foreign bodies can cause significant injuries to the oral cavity and gastrointestinal tract.	2A Major	Yes	3 Occasional. The danger has been observed and detected before as described [44]	5 Very severe. Foreign bodies can cause significant injuries to the oral cavity and gastrointestinal tract.	1 Existing detection measures. At the end of the process there is a metal detector and an X-ray machine for the detection of	15	Yes<br>S=5

Stage	Hazard	Control measures	4x4 Matrix model				FMEA model				
			Probability	Severity	Risk	SIG	Probability	Severity	Detection	CI	SIG
									any foreign bodies		
	(Q) Equipment chemicals and remains of cleaning and disinfection products	Preventative maintenance<br><br>Cleaning and disinfection plan<br><br>Training plan	1 Unlikely -to occur; may assume hazard will not occur due to the correct implementation of the PPRs for maintenance, cleaning and disinfection and staff training [58]	D Minor. The potential contamination would be residual and products used are authorized [57].	1D Minor	No	2 Unlikely These correspond to extremely isolated incidents due to the correct implementation of the PPRs for maintenance, cleaning and disinfection and staff training [58]	2 Mild. The potential contamination would be residual and products used are authorized [57].	5 There are no detection measures	20	No
	(B) Microorganisms (*Clostridium, Staphylococcus, Pseudomonas, Enterobacteriaceae*)	Control of pH (<4,3) and NaCl (4-5%)	2 Possible to occur in time if not corrected [64]	A Severe. Imminent and immediate danger of death or severe illness as described [40]	2A Major	Yes	3 Occasional. The danger has been observed and detected before as described [64]	5 Very severe. Failure in the process potentially affects food safety. It involves legal breaches or damage to	1 Existing detection measures. pH and salt are monitored.	15	Yes<br><br>S=5

Stage	Hazard	Control measures	4x4 Matrix model				FMEA model				
			Probability	Severity	Risk	SIG	Probability	Severity	Detection	CI	SIG
								the consumer's health as described [40]			
17.- Fruit reception and discharge	(P) Foreign bodies	Preventative maintenance	2 Possible to occur in time if not corrected [44]	A Severe. Foreign bodies can cause significant injuries to the oral cavity and gastro-intestinal tract.	2A Major	Yes	3 Occasional. The danger has been observed and detected before as described [44]	5 Very severe. Foreign bodies can cause significant injuries to the oral cavity and gastrointestinal tract.	1 Existing detection measures. At the end of the process there is a metal detector and an X-ray machine for the detection of any foreign bodies	15	Yes<br>S=5
	(Q) Remains of cleaning and disinfection products	Cleaning and disinfection plan<br>Training plan	1 Unlikely -to occur; may assume hazard will not occur due to the correct implementation of the PPRs for cleaning and disinfection	D Minor. The potential contamination would be residual and products used are authorized [57].	1D Minor	No	2 Unlikely These correspond to extremely isolated incidents due to the correct implementation of the PPRs for cleaning and	2 Mild. The potential contamination would be residual and products used are authorized [57].	5 There are no detection measures	20	No

Stage	Hazard	Control measures	4x4 Matrix model				FMEA model				
			Probability	Severity	Risk	SIG	Probability	Severity	Detection	CI	SIG
			and staff training [58]				disinfection and staff training [58]				
	(B) Microorganisms (*Clostridium, Staphylococcus, Pseudomonas, Enterobacteriaceae*)	Control of pH and NaCl<br><br>Cleaning and disinfection plan	2 Possible to occur in time if not corrected [64]	A Severe. Imminent and immediate danger of death or severe illness as described [40]	2A Major	Yes	3 Occasional. The danger has been observed and detected before as described [64]	5 Very severe. Failure in the process potentially affects food safety. It involves legal breaches or damage to the consumer's health as described [40]	1 Existing detection measures. pH and salt are monitored.	15	Yes<br><br>S=5
18.- Discharge hopper	(P) Foreign bodies	Preventative maintenance	2 Possible to occur in time if not corrected [44]	A Severe. Foreign bodies can cause significant injuries to the oral cavity and gastrointestinal tract.	2A Major	Yes	3 Occasional. The danger has been observed and detected before as described [44]	5 Very severe. Foreign bodies can cause significant injuries to the oral cavity and gastrointestinal tract.	1 Existing detection measures. At the end of the process there is a metal detector and an X-ray machine for the detection of	15	Yes<br><br>S=5

https://doi.org/10.3390/xxxxx

Stage	Hazard	Control measures	4x4 Matrix model				FMEA model				
			Probability	Severity	Risk	SIG	Probability	Severity	Detection	CI	SIG
									any foreign bodies		
	(C) Equipment chemicals and remains of cleaning and disinfection products	Preventative maintenance<br><br>Cleaning and disinfection plan<br><br>Training plan	1 Unlikely -to occur; may assume hazard will not occur due to the correct implementation of the PPRs for maintenance, cleaning and disinfection and staff training [58]	D Minor. The potential contamination would be residual and products used are authorized [57].	1D Minor	No	2 Unlikely These correspond to extremely isolated incidents due to the correct implementation of the PPRs for maintenance, cleaning and disinfection and staff training [58]	2 Mild. The potential contamination would be residual and products used are authorized [57].	5 There are no detection measures	20	No
	(B) Microorganisms (mainly mesophilic aerobes)	Cleaning and disinfection plan	1 Unlikely to occur; may assume hazard will not occur due to the correct implementation of the PPRs for cleaning and	D Minor. The potential contamination would be residual.	1D Minor	No	2 Unlikely. These correspond to extremely isolated incidents due to the correct implementation of the PPRs for	2 Mild. The potential contamination would be residual.	5 There are no detection measures	20	No

Stage	Hazard	Control measures	4x4 Matrix model				FMEA model				
			Probability	Severity	Risk	SIG	Probability	Severity	Detection	CI	SIG
			disinfection and staff training.				cleaning and disinfection and staff training.				
19.- Elevator and transportation (pipelines)	(P) Foreign bodies	Preventative maintenance	2 Possible to occur in time if not corrected [44]	A Severe. Foreign bodies can cause significant injuries to the oral cavity and gastrointestinal tract.	2A Major	Yes	3 Occasional. The danger has been observed and detected before as described [44]	5 Very severe. Foreign bodies can cause significant injuries to the oral cavity and gastrointestinal tract.	1 Existing detection measures. At the end of the process there is a metal detector and an X-ray machine for the detection of any foreign bodies	15	Yes S=5
	(C) Equipment chemicals and remains of cleaning and disinfection products	Preventative maintenance<br>Cleaning and disinfection plan<br>Training plan	1 Unlikely -to occur; may assume hazard will not occur due to the correct implementation of the PPRs for maintenance, cleaning and disinfection	D Minor. The potential contamination would be residual and products used are authorized [57].	1D Minor	No	2 Unlikely These correspond to extremely isolated incidents due to the correct implementation of the PPRs for maintenance, cleaning and	2 Mild. The potential contamination would be residual and products used are authorized [57].	5 There are no detection measures	20	No

Stage	Hazard	Control measures	4x4 Matrix model				FMEA model				
			Probability	Severity	Risk	SIG	Probability	Severity	Detection	CI	SIG
			and staff training [58]				disinfection and staff training [58]				
	(B) Microorganisms (mainly mesophilic aerobes)	Cleaning and disinfection plan<br><br>Training plan	1 Unlikely to occur; may assume hazard will not occur due to the correct implementation of the PPRs for cleaning and disinfection and staff training.	D Minor. The potential contamination would be residual.	1D Minor	No	2 Unlikely. These correspond to extremely isolated incidents due to the correct implementation of the PPRs for cleaning and disinfection and staff training.	2 Mild. The potential contamination would be residual.	5 There are no detection measures	20	No
20.- Nurse tank	(P) Foreign bodies	Preventative maintenance	2 Possible to occur in time if not corrected [44]	A Severe. Foreign bodies can cause significant injuries to the oral cavity and gastro-intestinal tract.	2A Major	Yes	3 Occasional. The danger has been observed and detected before as described [44]	5 Very severe. Foreign bodies can cause significant injuries to the oral cavity and gastrointestinal tract.	1 Existing detection measures. At the end of the process there is a metal detector and an X-ray machine for the detection of	15	Yes<br><br>S=5

Stage	Hazard	Control measures	4x4 Matrix model				FMEA model				
			Probability	Severity	Risk	SIG	Probability	Severity	Detection	CI	SIG
									any foreign bodies		
	(C) Equipment chemicals and remains of cleaning and disinfection products	Preventative maintenance<br><br>Cleaning and disinfection plan<br><br>Training plan	1 Unlikely -to occur; may assume hazard will not occur due to the correct implementation of the PPRs for maintenance, cleaning and disinfection and staff training [58]	D Minor. The potential contamination would be residual and products used are authorized [57].	1D Minor	No	2 Unlikely These correspond to extremely isolated incidents due to the correct implementation of the PPRs for maintenance, cleaning and disinfection and staff training [58]	2 Mild. The potential contamination would be residual and products used are authorized [57].	5 There are no detection measures	20	No
	(B) Microorganisms (mainly mesophilic aerobes)	Cleaning and disinfection plan<br><br>Training plan	1 Unlikely to occur; may assume hazard will not occur due to the correct implementation of the PPRs for cleaning and	D Minor. The potential contamination would be residual.	1D Minor	No	2 Unlikely. These correspond to extremely isolated incidents due to the correct implementation of the PPRs for	2 Mild. The potential contamination would be residual.	5 There are no detection measures	20	No

**Stage**	**Hazard**	**Control measures**	**4x4 Matrix model**				**FMEA model**				
			**Probability**	**Severity**	**Risk**	**SIG**	**Probability**	**Severity**	**Detection**	**CI**	**SIG**
			disinfection and staff training.				cleaning and disinfection and staff training.				
21.- Feed lines /distribution belt	(P) Foreign bodies	Preventative maintenance	2 Possible to occur in time if not corrected [44]	A Severe. Foreign bodies can cause significant injuries to the oral cavity and gastro-intestinal tract.	2A Major	Yes	3 Occasional. The danger has been observed and detected before as described [44]	5 Very severe. Foreign bodies can cause significant injuries to the oral cavity and gastrointestinal tract.	1 Existing detection measures. At the end of the process there is a metal detector and an X-ray machine for the detection of any foreign bodies	15	Yes S=5
	(C) Equipment chemicals and remains of cleaning and disinfection products	Preventative maintenance<br>Cleaning and disinfection plan<br>Training plan	1 Unlikely -to occur; may assume hazard will not occur due to the correct implementation of the PPRs for maintenance, cleaning and disinfection	D Minor. The potential contamination would be residual and products used are authorized [57].	1D Minor	No	2 Unlikely These correspond to extremely isolated incidents due to the correct implementation of the PPRs for maintenance, cleaning and	2 Mild. The potential contamination would be residual and products used are authorized [57].	5 There are no detection measures	20	No

Stage	Hazard	Control measures	4x4 Matrix model				FMEA model				
			Probability	Severity	Risk	SIG	Probability	Severity	Detection	CI	SIG
			and staff training [58]				disinfection and staff training [58]				
	(B) Microorganisms (mainly mesophilic aerobes)	Cleaning and disinfection plan<br><br>Training plan	1 Unlikely to occur; may assume hazard will not occur due to the correct implementation of the PPRs for cleaning and disinfection and staff training.	D Minor. The potential contamination would be residual.	1D Minor	No	2 Unlikely. These correspond to extremely isolated incidents due to the correct implementation of the PPRs for cleaning and disinfection and staff training.	2 Mild. The potential contamination would be residual.	5 There are no detection measures	20	No
22.- Pitting machine	(P) Foreign bodies (including pits)	Preventative maintenance	2 Possible to occur in time if not corrected [44]	A Severe. Foreign bodies can cause significant injuries to the oral cavity and	2A Major	Yes	3 Occasional. The danger has been observed and detected before as described [44]	5 Very severe. Foreign bodies can cause significant injuries to the oral cavity and	1 Existing detection measures. At the end of the process there is a metal detector and an X-ray	15	Yes<br><br>S=5

Stage	Hazard	Control measures	4x4 Matrix model				FMEA model				
			Probability	Severity	Risk	SIG	Probability	Severity	Detection	CI	SIG
				gastrointestinal tract.				gastrointestinal tract.	machine for the detection of any foreign bodies		
	(C) Equipment chemicals and remains of cleaning and disinfection products	Preventative maintenance<br><br>Cleaning and disinfection plan<br><br>Training plan	1 Unlikely -to occur; may assume hazard will not occur due to the correct implementation of the PPRs for maintenance, cleaning and disinfection and staff training [58]	D Minor. The potential contamination would be residual and products used are authorized [57].	1D Minor	No	2 Unlikely These correspond to extremely isolated incidents due to the correct implementation of the PPRs for maintenance, cleaning and disinfection and staff training [58]	2 Mild. The potential contamination would be residual and products used are authorized [57].	5 There are no detection measures	20	No
	(B) Microorganisms (mainly mesophilic aerobes)	Cleaning and disinfection plan<br><br>Training plan	1 Unlikely to occur; may assume hazard will not occur due to the correct implementation of the PPRs for	D Minor. The potential contamination would be residual.	1D Minor	No	2 Unlikely. These correspond to extremely isolated incidents due to the correct implementation of the	2 Mild. The potential contamination would be residual.	5 There are no detection measures	20	No

**Stage**	**Hazard**	**Control measures**	**4x4 Matrix model**				**FMEA model**				
			**Probability**	**Severity**	**Risk**	**SIG**	**Probability**	**Severity**	**Detection**	**CI**	**SIG**
			cleaning and disinfection and staff training.				PPRs for cleaning and disinfection and staff training.				
23.- Exit belt	(P) Foreign bodies	Preventative maintenance	2 Possible to occur in time if not corrected [44]	A Severe. Foreign bodies can cause significant injuries to the oral cavity and gastro-intestinal tract.	2A Major	Yes	3 Occasional. The danger has been observed and detected before as described [44]	5 Very severe. Foreign bodies can cause significant injuries to the oral cavity and gastrointestinal tract.	1 Existing detection measures. At the end of the process there is a metal detector and an X-ray machine for the detection of any foreign bodies	15	Yes S=5
	(C) Equipment chemicals and remains of cleaning and disinfection products	Preventative maintenance<br>Cleaning and disinfection plan<br>Training plan	1 Unlikely to occur; may assume hazard will not occur due to the correct implementation of the PPRs for maintenance, cleaning and	D Minor. The potential contamination would be residual and products used are authorized [57].	1D Minor	No	2 Unlikely These correspond to extremely isolated incidents due to the correct implementation of the PPRs for maintenance,	2 Mild. The potential contamination would be residual and products used are authorized [57].	5 There are no detection measures	20	No

Stage	Hazard	Control measures	4x4 Matrix model				FMEA model				
			Probability	Severity	Risk	SIG	Probability	Severity	Detection	CI	SIG
			disinfection and staff training [58]				cleaning and disinfection and staff training [58]				
	(B) Microorganisms (mainly mesophilic aerobes)	Cleaning and disinfection plan<br>Training plan	1 Unlikely to occur; may assume hazard will not occur due to the correct implementation of the PPRs for cleaning and disinfection and staff training.	D Minor. The potential contamination would be residual.	1D Minor	No	2 Unlikely. These correspond to extremely isolated incidents due to the correct implementation of the PPRs for cleaning and disinfection and staff training.	2 Mild. The potential contamination would be residual.	5 There are no detection measures	20	No
24.- Transport channels	(P) Foreign bodies	Preventative maintenance	2 Possible to occur in time if not corrected [44]	A Severe. Foreign bodies can cause significant injuries to the oral cavity and gastrointestinal tract.	2A Major	Yes	3 Occasional. The danger has been observed and detected before as described [44]	5 Very severe. Foreign bodies can cause significant injuries to the oral cavity and gastrointestinal tract.	1 Existing detection measures. At the end of the process there is a metal detector and an X-ray machine for the detection of	15	Yes<br>S=5

Stage	Hazard	Control measures	4x4 Matrix model				FMEA model				
			Probability	Severity	Risk	SIG	Probability	Severity	Detection	CI	SIG
									any foreign bodies		
	(C) Equipment chemicals and remains of cleaning and disinfection products<br><br>Heavy metals of water	Preventative maintenance<br><br>Cleaning and disinfection plan<br><br>Water control plan	2 Possible to occur in time if not corrected [58, 50]	B Severe not imminent as described [40, 45, 47, 48, 49]	2B Medium	Yes	3 Occasional. The danger has been observed and detected before as described [58,50]	5 Very severe. Failure in the process potentially affects food safety. It involves legal breaches or damage to the consumer's health as described [40, 45, 47, 48, 49]	1 Existing detection measures. Water plan includes analysis of these hazards.	15	Yes<br><br>S=5
	(B) Microorganisms	Cleaning and disinfection plan<br><br>Water plan control	2 Possible to occur in time if not corrected [40]	A Severe. Imminent and immediate danger of death or severe illness as described [40]	2A Major	Yes	3 Occasional. The danger has been observed and detected before [40].	5 Very severe. Failure in the process potentially affects food safety. It involves legal breaches or	1 Existing detection measures. Water control plan includes analysis to determine	15	Yes<br><br>S=5

Stage	Hazard	Control measures	4x4 Matrix model				FMEA model				
			**Probability**	**Severity**	**Risk**	**SIG**	**Probability**	**Severity**	**Detection**	**CI**	**SIG**
								damage to the consumer's health as described [40]			
25.- Accumulation hopper	(P) Foreign bodies	Preventative maintenance	2 Possible to occur in time if not corrected [44]	A Severe. Foreign bodies can cause significant injuries to the oral cavity and gastro-intestinal tract.	2A Major	Yes	3 Occasional. The danger has been observed and detected before as described [44]	5 Very severe. Foreign bodies can cause significant injuries to the oral cavity and gastrointestinal tract.	1 Existing detection measures. At the end of the process there is a metal detector and an X-ray machine for the detection of any foreign bodies	15	Yes S=5
	(C) Equipment chemicals and remains of cleaning and disinfection products	Preventative maintenance<br>Cleaning and disinfection plan<br>Training plan	1 Unlikely to occur; may assume hazard will not occur due to the correct implementation of the PPRs for maintenance,	D Minor. The potential contamination would be residual and products used are authorized [57].	1D Minor	No	2 Unlikely These correspond to extremely isolated incidents due to the correct implementation of the PPRs for	2 Mild. The potential contamination would be residual and products used are authorized [57].	5 There are no detection measures	20	No

Stage	Hazard	Control measures	4x4 Matrix model				FMEA model				
			Probability	Severity	Risk	SIG	Probability	Severity	Detection	CI	SIG
			cleaning and disinfection and staff training [58]				maintenance, cleaning and disinfection and staff training [58]				
	(B) Microorganisms (mainly mesophilic aerobes)	Cleaning and disinfection plan<br>Training plan	1 Unlikely to occur; may assume hazard will not occur due to the correct implementation of the PPRs for cleaning and disinfection and staff training.	D Minor. The potential contamination would be residual.	1D Minor	No	2 Unlikely. These correspond to extremely isolated incidents due to the correct implementation of the PPRs for cleaning and disinfection and staff training.	2 Mild. The potential contamination would be residual.	5 There are no detection measures	20	No
26.- Elevator	(P) Foreign bodies (including pits)	Preventative maintenance	2 Possible to occur in time if not corrected [44]	A Severe. Foreign bodies can cause significant injuries to the oral cavity and	2A Major	Yes	3 Occasional. The danger has been observed and detected before as described [44]	5 Very severe. Foreign bodies can cause significant injuries to the oral cavity and	1 Existing detection measures. At the end of the process there is a metal detector and an X-ray machine for the	15	Yes<br>S=5

Stage	Hazard	Control measures	4x4 Matrix model				FMEA model				
			**Probability**	**Severity**	**Risk**	**SIG**	**Probability**	**Severity**	**Detection**	**CI**	**SIG**
				gastrointestinal tract.				gastrointestinal tract.	detection of any foreign bodies		
	(C) Equipment chemicals and remains of cleaning and disinfection products	Preventative maintenance<br><br>Cleaning and disinfection plan<br><br>Training plan	1 Unlikely to occur; may assume hazard will not occur due to the correct implementation of the PPRs for maintenance, cleaning and disinfection and staff training [58]	D Minor. The potential contamination would be residual and products used are authorized [57].	1D Minor	No	2 Unlikely These correspond to extremely isolated incidents due to the correct implementation of the PPRs for maintenance, cleaning and disinfection and staff training [58]	2 Mild. The potential contamination would be residual and products used are authorized [57].	5 There are no detection measures	20	No
	(B) Microorganisms (mainly mesophilic aerobes)	Cleaning and disinfection plan<br><br>Training plan	1 Unlikely to occur; may assume hazard will not occur due to the correct implementation of the PPRs for cleaning and	D Minor. The potential contamination would be residual.	1D Minor	No	2 Unlikely. These correspond to extremely isolated incidents due to the correct implementation of the PPRs for	2 Mild. The potential contamination would be residual.	5 There are no detection measures	20	No

Stage	Hazard	Control measures	4x4 Matrix model				FMEA model				
			Probability	Severity	Risk	SIG	Probability	Severity	Detection	CI	SIG
			disinfection and staff training.				cleaning and disinfection and staff training.				
27.- Flotation/ vibrator tank	(P) Foreign bodies	Preventative maintenance	2 Possible to occur in time if not corrected [44]	A Severe. Foreign bodies can cause significant injuries to the oral cavity and gastrointestinal tract.	2A Major	Yes	3 Occasional. The danger has been observed and detected before as described [44]	5 Very severe. Foreign bodies can cause significant injuries to the oral cavity and gastrointestinal tract.	1 Existing detection measures. At the end of the process there is a metal detector and an X-ray machine for the detection of any foreign bodies	15	Yes S=5
	(C) Equipment chemicals and remains of cleaning and disinfection products<br><br>Heavy metals of water	Preventative maintenance<br><br>Cleaning and disinfection plan<br><br>Water control plan	2 Possible to occur in time if not corrected [58, 50]	B Severe not imminent as described [40, 45, 47, 48, 49]	2B Medium	Yes	3 Occasional. The danger has been observed and detected before as described [58,50]	5 Very severe. Failure in the process potentially affects food safety. It involves legal breaches or damage to the consumer's	1 Existing detection measures. Water plan includes anlysis of these hazards.	15	Yes S=5

Stage	Hazard	Control measures	4x4 Matrix model				FMEA model				
			Probability	Severity	Risk	SIG	Probability	Severity	Detection	CI	SIG
								health as described [40, 45, 47, 48, 49]			
	(B) Microorganisms	Cleaning and disinfection plan<br><br>Water plan control	2 Possible to occur in time if not corrected [40]	A Severe. Imminent and immediate danger of death or severe illness as described [40]	2A Major	Yes	3 Occasional. The danger has been observed and detected before [40].	5 Very severe. Failure in the process potentially affects food safety. It involves legal breaches or damage to the consumer's health as described [40]	1 Existing detection measures	15	Yes<br><br>S=5
28.- Fruit selection	(P) Foreign bodies (including pits)	Preventative maintenance	2 Possible to occur in time if not corrected [44]	A Severe. Foreign bodies can cause significant injuries to the oral cavity and	2A Major	Yes	3 Occasional. The danger has been observed and detected before as described [44]	5 Very severe. Foreign bodies can cause significant injuries to the oral cavity and	1 Existing detection measures. At the end of the process there is a metal detector and an X-ray machine for the	15	Yes<br>S=5

Stage	Hazard	Control measures	4x4 Matrix model				FMEA model				
			Probability	Severity	Risk	SIG	Probability	Severity	Detection	CI	SIG
				gastrointestinal tract.				gastrointestinal tract.	detection of any foreign bodies		
	(C) Equipment chemicals and remains of cleaning and disinfection products	Preventative maintenance<br><br>Cleaning and disinfection plan<br><br>Training plan	1 Unlikely to occur; may assume hazard will not occur due to the correct implementation of the PPRs for maintenance, cleaning and disinfection and staff training [58]	D Minor. The potential contamination would be residual and products used are authorized [57].	1D Minor	No	2 Unlikely These correspond to extremely isolated incidents due to the correct implementation of the PPRs for maintenance, cleaning and disinfection and staff training [58]	2 Mild. The potential contamination would be residual and products used are authorized [57].	5 There are no detection measures	20	No
	(B) Microorganisms (mainly mesophilic aerobes)	Cleaning and disinfection plan<br><br>Training plan	1 Unlikely to occur; may assume hazard will not occur due to the correct implementation of the PPRs for cleaning and	D Minor. The potential contamination would be residual.	1D Minor	No	2 Unlikely. These correspond to extremely isolated incidents due to the correct implementation of the PPRs for	2 Mild. The potential contamination would be residual.	5 There are no detection measures	20	No

Stage	Hazard	Control measures	4x4 Matrix model Probability	4x4 Matrix model Severity	4x4 Matrix model Risk	4x4 Matrix model SIG	FMEA model Probability	FMEA model Severity	FMEA model Detection	FMEA model CI	FMEA model SIG
			disinfection and staff training.				cleaning and disinfection and staff training.				
29.- Metal detection	(P) Foreign metal bodies	Metal detection	3 Probably will occur in time if not corrected [44]	A Severe. Foreign bodies can cause significant injuries to the oral cavity and gastrointestinal tract.	3A Major	Yes	3 Occasional. The danger has been observed and detected before as described [44]	5 Very severe. Foreign bodies can cause significant injuries to the oral cavity and gastrointestinal tract.	1 Existing detection measures. The metal detector is itself a measure for detecting hazards of metallic origin.	15	Yes S=5
	(C) Remains of cleaning and disinfection products	Cleaning and disinfection plan Training plan	1 Unlikely to occur; may assume hazard will not occur due to the correct implementation of the PPRs for cleaning and disinfection and staff training [58]	D Minor. The potential contamination would be residual and products used are authorized [57].	1D Minor	No	2 Unlikely These correspond to extremely isolated incidents due to the correct implementation of the PPRs for cleaning and disinfection and staff training [58]	2 Mild. The potential contamination would be residual and products used are authorized [57].	5 There are no detection measures	20	No

Stage	Hazard	Control measures	4x4 Matrix model				FMEA model				
			Probability	Severity	Risk	SIG	Probability	Severity	Detection	CI	SIG
	(B) Microorganisms (mainly mesophilic aerobes)	Cleaning and disinfection plan<br><br>Training plan	1 Unlikely. to occur; may assume" hazard will not occur according to organization's history.	D Minor. Illness or injury is minor.	1D Minor	No	2 Unlikely. These correspond to extremely isolated incidents according to organization's history.	2 Mild. The safety of the final product is not affected,	5 There are no detection measures	20	No
30.-Accumulation hopper	(P) Foreign bodies	Preventative maintenance	2 Possible to occur in time if not corrected [44]	A Severe. Foreign bodies can cause significant injuries to the oral cavity and gastrointestinal tract.	2A Major	Yes	3 Occasional. The danger has been observed and detected before as described [44]	5 Very severe. Foreign bodies can cause significant injuries to the oral cavity and gastrointestinal tract.	1 Existing detection measures. At the end of the process there is an X-ray machine for the detection of any foreign bodies	15	Yes<br><br>S=5
	(C) Equipment chemicals and remains of cleaning and disinfection products	Preventative maintenance<br><br>Cleaning and disinfection plan<br><br>Training plan	1 Unlikely to occur; may assume hazard will not occur due to the correct implementation of the	D Minor. The potential contamination would be residual and products used	1D Minor	No	2 Unlikely These correspond to extremely isolated incidents due to the correct	2 Mild. The potential contamination would be residual and products used	5 There are no detection measures	20	No

Stage	Hazard	Control measures	4x4 Matrix model				FMEA model				
			Probability	Severity	Risk	SIG	Probability	Severity	Detection	CI	SIG
			PPRs for maintenance, cleaning and disinfection and staff training [58]	are authorized [57].			implementation of the PPRs for maintenance, cleaning and disinfection and staff training [58]	are authorized [57].			
	(B) Microorganisms (mainly mesophilic aerobes)	Cleaning and disinfection plan<br><br>Training plan	1 Unlikely to occur; may assume hazard will not occur due to the correct implementation of the PPRs for cleaning and disinfection and staff training.	D Minor. The potential contamination would be residual.	1D Minor	No	2 Unlikely. These correspond to extremely isolated incidents due to the correct implementation of the PPRs for cleaning and disinfection and staff training.	2 Mild. The potential contamination would be residual.	5 There are no detection measures	20	No
31.- Elevator and transport	(P) Foreign bodies	Preventative maintenance	2 Possible to occur in time if not corrected [44]	A Severe. Foreign bodies can cause significant injuries to the	2A Major	Yes	3 Occasional. The danger has been observed and detected be-	5 Very severe. Foreign bodies can cause significant injuries to	1 Existing detection measures. At the end of the process there is an X-ray	15	Yes<br><br>S=5

Stage	Hazard	Control measures	4x4 Matrix model				FMEA model				
			Probability	Severity	Risk	SIG	Probability	Severity	Detection	CI	SIG
				oral cavity and gastro-intestinal tract.			fore as described [44]	the oral cavity and gastrointestinal tract.	machine for the detection of any foreign bodies		
	(C) Equipment chemicals and remains of cleaning and disinfection products	Preventative maintenance<br><br>Cleaning and disinfection plan<br><br>Training plan	1 Unlikely to occur; may assume hazard will not occur due to the correct implementation of the PPRs for maintenance, cleaning and disinfection and staff training [58]	D Minor. The potential contamination would be residual and products used are authorized [57].	1D Minor	No	2 Unlikely These correspond to extremely isolated incidents due to the correct implementation of the PPRs for maintenance, cleaning and disinfection and staff training [58]	2 Mild. The potential contamination would be residual and products used are authorized [57].	5 There are no detection measures	20	No
	(B) Microorganisms (mainly mesophilic aerobes)	Cleaning and disinfection plan<br><br>Training plan	1 Unlikely to occur; may assume hazard will not occur due to the correct implementation of the PPRs for	D Minor. The potential contamination would be residual.	1D Minor	No	2 Unlikely. These correspond to extremely isolated incidents due to the correct implementation of the	2 Mild. The potential contamination would be residual.	5 There are no detection measures	20	No

Stage	Hazard	Control measures	4x4 Matrix model				FMEA model				
			**Probability**	**Severity**	**Risk**	**SIG**	**Probability**	**Severity**	**Detection**	**CI**	**SIG**
			cleaning and disinfection and staff training.				PPRs for cleaning and disinfection and staff training.				
32.- Filling/ weighing	(P) Foreign bodies	Preventative maintenance<br>Good practices with containers	2 Possible to occur in time if not corrected [44]	A Severe. Foreign bodies can cause significant injuries to the oral cavity and gastrointestinal tract.	2A Major	Yes	3 Occasional. The danger has been observed and detected before as described [44]	5 Very severe. Foreign bodies can cause significant injuries to the oral cavity and gastrointestinal tract.	1 Existing detection measures. At the end of the process there is an X-ray machine for the detection of any foreign bodies.	15	Yes<br>S=5
	(C) Equipment chemicals and remains of cleaning and disinfection products	Preventative maintenance<br>Cleaning and disinfection plan<br>Training plan	1 Unlikely to occur; may assume hazard will not occur due to the correct implementation of the PPRs for maintenance, cleaning and disinfection	D Minor. The potential contamination would be residual and products used are authorized [57].	1D Minor	No	2 Unlikely These correspond to extremely isolated incidents due to the correct implementation of the PPRs for maintenance, cleaning and disinfection	2 Mild. The potential contamination would be residual and products used are authorized [57].	5 There are no detection measures	20	No

Stage	Hazard	Control measures	4x4 Matrix model				FMEA model				
			Probability	Severity	Risk	SIG	Probability	Severity	Detection	CI	SIG
			and staff training [58]				and staff training [58]				
	(B) Microorganisms (mainly mesophilic aerobes)	Cleaning and disinfection plan<br><br>Training plan	1 Unlikely to occur; may assume hazard will not occur due to the correct implementation of the PPRs for cleaning and disinfection and staff training.	D Minor. The potential contamination would be residual.	1D Minor	No	2 Unlikely. These correspond to extremely isolated incidents due to the correct implementation of the PPRs for cleaning and disinfection and staff training.	2 Mild. The potential contamination would be residual.	5 There are no detection measures	20	No
33.- Brine addition	(P) Foreign bodies	Preventative maintenance	2 Possible to occur in time if not corrected [44]	A Severe. Foreign bodies can cause significant injuries to the oral cavity and gastrointestinal tract.	2A Major	Yes	3 Occasional. The danger has been observed and detected before as described [44]	5 Very severe. Foreign bodies can cause significant injuries to the oral cavity and gastrointestinal tract.	1 Existing detection measures. At the end of the process there is an X-ray machine for the detection of any foreign bodies	15	Yes<br><br>S=5

Stage	Hazard	Control measures	4x4 Matrix model				FMEA model				
			**Probability**	**Severity**	**Risk**	**SIG**	**Probability**	**Severity**	**Detection**	**CI**	**SIG**
	(C) Equipment chemicals and remains of cleaning and disinfection products	Preventative maintenance<br><br>Cleaning and disinfection plan<br><br>Training plan	1 Unlikely - to occur; may assume hazard will not occur due to the correct implementation of the PPRs for maintenance, cleaning and disinfection and staff training [58]	D Minor. The potential contamination would be residual and products used are authorized [57].	1D Minor	No	2 Unlikely These correspond to extremely isolated incidents due to the correct implementation of the PPRs for maintenance, cleaning and disinfection and staff training [58]	2 Mild. The potential contamination would be residual and products used are authorized [57].	5 There are no detection measures	20	No
	(B) Microorganisms (mainly mesophilic aerobes)	Cleaning and disinfection plan<br><br>Control of the brine [Nacl (4-5%), pH (<4,3) and acidity (0,3-0,5%)].	2 Possible to occur in time if not corrected [64]	A Severe. Imminent and immediate danger of death or severe illness	2A Major	Yes	3 Occasional. The danger has been observed and detected before as described [64]	5 Very severe. Failure in the process potentially affects food safety. It involves legal breaches or damage to the	1 Existing detection measures. Control of salt, pH and acidity.	15	Yes<br><br>S=5

Stage	Hazard	Control measures	4x4 Matrix model				FMEA model				
			Probability	Severity	Risk	SIG	Probability	Severity	Detection	CI	SIG
								consumer's health.			
34.- Container sealing	(P) Foreign bodies	Preventative maintenance	2 Possible to occur in time if not corrected [44]	A Severe. Foreign bodies can cause significant injuries to the oral cavity and gastro-intestinal tract.	2A Major	Yes	3 Occasional. The danger has been observed and detected before as described [44]	5 Very severe. Foreign bodies can cause significant injuries to the oral cavity and gastrointestinal tract.	1 Existing detection measures. At the end of the process there is an X-ray machine for the detection of any foreign bodies	15	Yes S=5
	(C) Equipment chemicals and remains of cleaning and disinfection products	Preventative maintenance<br>Cleaning and disinfection plan<br>Training plan	1 Unlikely - to occur; may assume hazard will not occur due to the correct implementation of the PPRs for preventive maintenance, cleaning and disinfection	D Minor. The potential contamination would be residual and products used are authorized [57].	1D Minor	No	2 Unlike. These correspond to extremely isolated incidents due to the correct implementation of the PPRs for maintenance, cleaning and disinfection	2 Mild. The potential contamination would be residual and products used are authorized [57].	5 There are no detection measures	20	No

Stage	Hazard	Control measures	4x4 Matrix model				FMEA model				
			Probability	Severity	Risk	SIG	Probability	Severity	Detection	CI	SIG
			and staff training [58]				and staff training [58]				
	(B) Microorganisms	Control of hermetic sealing	2 Minor. The danger has been observed and detected before as described [65, 40]	A Severe. Imminent and immediate danger of death or severe illness as described [40]	2A Major	Yes	3 Occasional. The danger has been observed and detected before as described [65,40]	5 Very severe. Failure in the process potentially affects food safety. It involves legal breaches or damage to the consumer's health as described [40]	1 Existing detection measures. Control of hermetic sealing.	15	Yes S=5
35.-Pasteurization	(B) Microorganisms (*Clostridium* spp., aerobic mesophiles, fungal spores)	Temperature: 70-85ºC<br>Time: 5-20 minutes<br>15 PU units	2 Possible to occur in time if not corrected [28,66]	A Severe. Imminent and immediate danger of death or severe illness as described [40]	2A Major	Yes	3 Occasional. The danger has been observed and detected before as described [28, 66]	5 Very severe. Failure in the process potentially affects food safety. It involves legal breaches or damage to	1 Existing detection measures. Control de temperature, time and pressure.	15	Yes S=5

Stage	Hazard	Control measures	4x4 Matrix model				FMEA model				
			Probability	Severity	Risk	SIG	Probability	Severity	Detection	CI	SIG
								the consumer's health as described [40]			
36.- Container drying	(P) Glass splinter	Good manufacturing practices	2 Possible to occur in time if not corrected [44]	A Severe. Foreign bodies can cause significant injuries to the oral cavity and gastrointestinal tract.	2A Major	Yes	3 Occasional. The danger has been observed and detected before as described [44]	5 Very severe. Foreign bodies can cause significant injuries to the oral cavity and gastrointestinal tract.	1 Existing detection measures. The next stage of the process there is an X-ray machine for the detection of any foreign bodies	15	Yes<br>S=5
37.- X-ray detection	(P) Foreign bodies	X-ray detection	3 Probably will occur in time if not corrected [44]	A Severe. Foreign bodies can cause significant injuries to the oral cavity and gastrointestinal tract.	3A Major	Yes	3 Occasional. The danger has been observed and detected before as described [44]	5 Very severe. Foreign bodies can cause significant injuries to the oral cavity and gastrointestinal tract.	1 Existing detection measures. The X-ray equipment is itself a measure for detecting foreign bodies.	15	Yes<br>S=5

Stage	Hazard	Control measures	4x4 Matrix model				FMEA model				
			Probability	Severity	Risk	SIG	Probability	Severity	Detection	CI	SIG
38.- Labelling	(P) Glass splinter	Good manufacturing practices	3 Probably. will occur in time if not corrected. The hazard has been recorded in the organization's history.	A Severe. Glass fragments can cause significant injuries to the oral cavity and gas-tro-intestinal tract.	3A Major	Yes	3 Occasional. The danger has been observed and detected before in the organization's history.	5 Very severe. Glass fragments can cause significant injuries to the oral cavity and gastrointestinal tract.	5 There are no detection measures	75	Yes
39.- Palletizing	(P) Glass splinter	Good manufacturing practices	3 Probably will occur in time if not corrected. The hazard has been recorded in the orga-ni-za-tion's history.	A Severe. Glass fragments can cause significant injuries to the oral cavity and gastro-intes-tinal tract.	3A Major	Yes	3 Occasional. The danger has been observed and detected before in the organization's history.	5 Very severe. Glass fragments can cause significant injuries to the oral cavity and gas-tro-intes-tinal tract.	5 There are no detection measures	75	Yes
40.- Storage	(P) Glass splinter	Good manufacturing practices	3 Probably will occur in time if not corrected. The hazard has been	A Severe. Glass fragments can cause significant injuries to the	3A Major	Yes	3 Occasional. The danger has been observed and detected before in the	5 Very severe. Glass fragments can cause significant injuries to	5 There are no detection measures	75	Yes

https://doi.org/10.3390/xxxxx

Stage	Hazard	Control measures	4x4 Matrix model				FMEA model				
			Probability	Severity	Risk	SIG	Probability	Severity	Detection	CI	SIG
			recorded in the organi-za-tion's history.	oral cavity and gastro-intes-tinal tract.			organiza-tion's history.	the oral cavity and gas-tro-intes-tinal tract.			
41.- Shipping	(P) Glass splinter	Good manufac-turing practices	3 Probably will occur in time if not corrected. The hazard has been rec-orded in the organiza-tion's history.	A Severe. Glass frag-ments can cause sig-nificant in-juries to the oral cavity and gastro-intes-tinal tract.	3A Major	Yes	3 Occasional. The danger has been ob-served and detected be-fore in the or-ganization's history.	5 Very se-vere. Glass fragments can cause significant injuries to the oral cavity and gastro-intes-tinal tract.	5 There are no detection measures	75	Yes

https://doi.org/10.3390/xxxxx

Table S5. CCP and stricter PRP determination after applying 4x4 matrix and FMEA model in Spanish-style olive processing

Stage/Hazard	4x4 Risk matrix	FMEA Risk method*	Q1	Q2	Q3	Q4	CCP/Stricter PRP
**1.- Olive receiving hopper**<br>(C) Heavy metals, pesticides and mycotoxins	Major	CI=15<br>S=5	Yes<br>(SAP, RP)	-	-	-	Stricter PRP
**1.- Olive receiving hopper**<br>(B) Microorganisms (*Escherichia coli, Salmonella, Clostridium…*)	Major	CI=15<br>S=5	Yes<br>(SAP, RP)	-	-	-	Stricter PRP
**2.- Reception of packaging and labelling materials**<br>(P) Foreign bodies	Major	CI=15<br>S=5	Yes<br>(SAP, RP)	-	-	-	Stricter PRP
**2.- Reception of packaging and labelling materials**<br>(C) Chemical migration	Medium	CI=10<br>S=5	Yes<br>(SAP, RP)	-	-	-	Stricter PRP
**3.- Reception of sodium hydroxide (NaOH) and salt (NaCl) reception**<br>(C) Incorrect chemical	Medium	CI=10<br>S=5	Yes<br>(SAP, RP)	-	-	-	Stricter PRP
**4.- Transportation elevator/conveyor belt**<br>(P) Foreign bodies	Major	CI=15<br>S=5	Yes<br>(PM)	-	-	-	Stricter PRP
**5.- Cleaner/**<br>**destemmer**<br>(P) Foreign bodies	Major	CI=15<br>S=5	Yes<br>(PM)	-	-	-	Stricter PRP

**Stage/Hazard**	**4x4 Risk matrix**	**FMEA Risk method***	**Q1**	**Q2**	**Q3**	**Q4**	**CCP/Stricter PRP**
**6.- Washing (I)** (P) Foreign bodies	Major	CI=15 S=5	Yes (PM)	-	-	-	Stricter PRP
**6.- Washing (I)** (C) Heavy metals	Medium	CI=15 S=5	Yes (WCP)	-	-	-	Stricter PRP
**6.- Washing (I)** Microorganisms (*Escherichia coli, Clostridium perfringens…*)	Major	CI=15 S=5	Yes (WCP)	-	-	-	Stricter PRP
**7.- Sorter** (P) Foreign bodies	Major	CI=15 S=5	Yes (PM)	-	-	-	Stricter PRP
**8.- Colour selector** (P) Foreign bodies	Major	CI=15 S=5	Yes (PM)	-	-	-	Stricter PRP
**9.- Transportation (elevator/ conveyor belt)** (P) Foreign bodies	Major	CI=15 S=5	Yes (PM)	-	-	-	Stricter PRP
**10.- Lye treatment**	Major	CI=15	Yes	-	-	-	Stricter PRP

Stage/Hazard	4x4 Risk matrix	FMEA Risk method*	Q1	Q2	Q3	Q4	CCP/Stricter PRP
(P) Foreign bodies		S=5	(PM)				
**11.-Washing (II)**<br>(P) Foreign bodies	Major	CI=15<br>S=5	Yes<br>(PM)	-	-	-	Stricter PRP
**11.- Washing (II)**<br>(C) Heavy metals	Medium	CI=15<br>S=5	Yes<br>(WCP)	-	-	-	Stricter PRP
**11.- Washing (II)**<br>Microorganisms (*Escherichia coli, Clostridium perfringens…*)	Major	CI=15<br>S=5	Yes<br>(WCP)	-	-	-	Stricter PRP
**12.- Brine placement**<br>(P) Foreign bodies	Major	CI=15<br>S=5	Yes<br>(PM)	-	-	-	Stricter PRP
**13.- Transport to fermentation vessels**<br>(P) Foreign bodies	Major	CI=15<br>S=5	Yes<br>(PM)	-	-	-	Stricter PRP
**14.- Fermentation**<br>(P) Foreign bodies	Major	CI=15<br>S=5	Yes<br>(PM)	-	-	-	Stricter PRP
**14.- Fermentation**	Major	CI=15	No	Yes	Yes	-	**CCP

Stage/Hazard	4x4 Risk matrix	FMEA Risk method*	Q1	Q2	Q3	Q4	CCP/Stricter PRP
(B) Microorganisms<br>(*Enterobacteriacea, Clostridium, Pseudomonas, Staphylococcus*, etc.)		S=5			(P)		
**15.- Storage in fermentation vessels**<br>(P) Foreign bodies	Major	CI=15<br>S=5	Yes<br>(PM)	-	-	-	Stricter PRP
**15.- Storage in fermentation vessels**<br>(B) Microorganisms<br>(*Enterobacteriaceae, Clostridium, Pseudomonas, Staphylococcus*, etc.)	Major	CI=15<br>S=5	No	Yes	Yes<br>(P)	-	**CCP
**16.- Fruit conditioning after storage**<br>(P) Foreign bodies	Major	CI=15<br>S=5	Yes<br>(PM)	-	-	-	Stricter PRP
**16.- Fruit conditioning after storage**<br>(B) Microorganisms<br>(*Enterobacteriaceae, Clostridium, Pseudomonas, Staphylococcus*, etc.)	Major	CI=15<br>S=5	No	Yes	Yes<br>(P)	-	**CCP
**17.- Fruit reception and discharge**<br>(P) Foreign bodies	Major	CI=15<br>S=5	Yes<br>(PM)	-	-	-	Stricter PRP
**17.- Fruit reception and discharge**<br>(B) Microorganisms	Major	CI=15<br>S=5	No	Yes	Yes<br>(P)	-	**CCP

**Stage/Hazard**	**4x4 Risk matrix**	**FMEA Risk method***	**Q1**	**Q2**	**Q3**	**Q4**	**CCP/Stricter PRP**
(*Enterobacteriaceae, Clostridium, Pseudomonas, Staphylococcus,* etc.)							
**18.- Discharge hopper** (P) Foreign bodies	Major	CI=15<br>S=5	Yes<br>(PM)	-	-	-	Stricter PRP
**19.- Elevator and transportation (pipelines)**<br>(P) Foreign bodies	Major	CI=15<br>S=5	Yes<br>(PM)	-	-	-	Stricter PRP
**20.- Nurse tank**<br>(P) Foreign bodies	Major	CI=15<br>S=5	Yes<br>(PM)	-	-	-	Stricter PRP
**21.- Feed lines**<br>**/Distribution belt**<br>(P) Foreign bodies	Major	CI=15<br>S=5	Yes<br>(PM)	-	-	-	Stricter PRP
**22.- Pitting machine**<br>(P) Foreign bodies (including pits)	Major	CI=15<br>S=5	Yes<br>(PM)	-	-	-	Stricter PRP
**23.- Exit belt**<br>(P) Foreign bodies	Major	CI=15<br>S=5	Yes<br>(PM)	-	-	-	Stricter PRP
**24.- Transport channels**<br>(P) Foreign bodies	Major	CI=15<br>S=5	Yes<br>(PM)	-	-	-	Stricter PRP

Stage/Hazard	4x4 Risk matrix	FMEA Risk method*	Q1	Q2	Q3	Q4	CCP/Stricter PRP
**24.- Transport channels**<br>(C) Heavy metals	Medium	CI=15<br>S=5	Yes<br>(WCP)	-	-	-	Stricter PRP
**24.- Transport channels**<br>(B) Microorganisms	Major	CI=15<br>S=5	Yes<br>(WCP)	-	-	-	Stricter PRP
**25.- Accumulation hopper**<br>(P) Foreign bodies	Major	CI=15<br>S=5	Yes<br>(PM)	-	-	-	Stricter PRP
**26.- Elevator**<br>(P) Foreign bodies	Major	CI=15<br>S=5	Yes<br>(PM)	-	-	-	Stricter PRP
**27.- Flotation/ vibrator tank**<br>(P) Foreign bodies	Major	CI=15<br>S=5	Yes<br>(PM)	-	-	-	Stricter PRP
**27.- Flotation/ vibrator tank**<br>(C) Heavy metals	Medium	CI=15<br>S=5	Yes<br>(WCP)	-	-	-	Stricter PRP

Stage/Hazard	4x4 Risk matrix	FMEA Risk method*	Q1	Q2	Q3	Q4	CCP/Stricter PRP
**27.- Flotation/ vibrator tank** (B) Microorganisms	Major	CI=15 S=5	Yes (WCP)	-	-	-	Stricter PRP
**28.- Fruit selection** (P) Foreign bodies	Major	CI=15 S=5	Yes (PM)	-	-	-	Stricter PRP
**29.- Metal detection** (P) Foreign metal bodies	Major	CI=15 S=5	No	Yes	No	Yes	CCP
**30.- Accumulation hopper** (P) Foreign bodies	Major	CI=15 S=5	Yes (PM)	-	-	-	Stricter PRP
**31.- Elevator and transport** (P) Foreign bodies	Major	CI=15 S=5	Yes (PM)	-	-	-	Stricter PRP
**32.- Filling/weighing** (P) Foreign bodies	Major	CI=15 S=5	Yes (PM)	-	-	-	Stricter PRP
**33.- Brine addition** (P) Foreign bodies	Major	CI=15 S=5	Yes (PM)	-	-	-	Stricter PRP
**33.- Brine addition**	Major	CI=15	No	Yes	Yes	-	**CCP

Stage/Hazard	4x4 Risk matrix	FMEA Risk method*	Q1	Q2	Q3	Q4	CCP/Stricter PRP
Microorganisms		S=5			(P)		
**34.- Container sealing** (P) Foreign bodies	Major	CI=15 S=5	Yes (PM)	-	-	-	Stricter PRP
**34.- Container sealing** (B) Microorganisms	Major	CI=15 S=5	No	Yes	Yes (P)	-	**CCP
**35.- Pasteurization** (B) Microorganisms (*Clostridium*, aerobic mesophiles, fungal spores)	Major	CI=15 S=5	No	Yes	No	Yes	CCP
**36.- Container drying** (P) Glass splinter	Major	CI=15 S=5	Yes (GMP)	-	-	-	Stricter PRP
**37.- X-ray detection** (P) Foreign bodies	Major	CI=15 S=5	No	Yes	No	Yes	CCP
**38.- Labelling** (P) Glass splinter	Major	CI=15 S=5	Yes (GMP)	-	-	-	Stricter PRP
**39.- Palletizing** (P) Glass splinter	Major	CI=15 S=5	Yes (GMP)	-	-	-	Stricter PRP
**40.- Storage**	Major	CI=15	Yes	-	-	-	Stricter PRP

Stage/Hazard	4x4 Risk matrix	FMEA Risk method*	Q1	Q2	Q3	Q4	CCP/Stricter PRP
(P) Glass splinter		S=5	(GMP)				
**41.- Shipping**<br>(P) Glass splinter	Major	CI=15<br>S=5	Yes<br>(GMP)	-	-	-	Stricter PRP

P=Physical hazard; C=Chemical hazard; B=Biological hazard; SAP= Supplier approval plan; RP= Reception plan; GMP=Good manufacturing practices; PZ=Pasteurization; WCP= Water control plan; PM=Preventive maintenance.

*Risk: CI= Criticality Index; S=severity.

**Consider whether the control measure at this step works in combination with a control measure at another step to control the same hazard, in which case both steps should be considered as CCPs.

Table S6. Hazard analysis after applying 4x4 matrix and FMEA model in specific stages of Californian-style black olive processing

Stage	Hazard	Control measures	4x4 matrix model				FMEA model				
			Probability	Severity	Risk	SIG	Probability	Severity	Detection	CI	SIG
I.- Storage in fiberglass tanks with acidified brine and air blowing	(P) Foreign bodies	Preventative maintenance	2 Possible to occur in time if not corrected [44]	A Severe Foreign bodies can cause significant injuries to the oral cavity and gastro-intestinal tract.	2A Major	Yes	3 Occasional The danger has been observed and detected before as described [44]	5 Very severe Foreign bodies can cause significant injuries to the oral cavity and gastrointestinal tract.	1 Existing detection measures. At the end of the process there is a metal detector and an X-ray machine for the detection of any foreign bodies	15	Yes S=5
	(C) Heavy metals	Water control plan	2 Possible to occur in time if not corrected [50]	B Severe not imminent as described [40, 45, 47, 48, 49]	2B Medium	Yes	3 Occasional The danger has been observed and detected before as described [50]	5 Very severe Failure in the process potentially affects food safety. It involves legal breaches or damage to the consumer's health as described	1 Existing detection measures Water control plan includes analysis to determine heavy metals	15	Yes S=5

Stage	Hazard	Control measures	4x4 matrix model				FMEA model				
			**Probability**	**Severity**	**Risk**	**SIG**	**Probability**	**Severity**	**Detection**	**CI**	**SIG**
								[40, 45, 47, 48, 49]			
	(B) Microorganisms (*Escherichia, Salmonella, Clostridium*…)	Control of pH (pH<4,3) and air blowing control.	2 Possible to occur in time if not corrected [76, 77]	A Severe Imminent and immediate danger of death or severe illness as described [40]	2A Major	Yes	3 Occasional The danger has been observed and detected before as described [76, 77]	5 Very severe Imminent and immediate dan-ger of death or severe illness as described [40]	1 Existing detection measures. Control of pH (pH<4,3) and air blowing control.	15	Yes S=5
II.- Oxidation, lye treatment, and color fixation in aeration tanks	(P) Foreign bodies	Preventative maintenance	2 Possible to occur in time if not corrected [44]	A Severe Foreign bodies can cause significant injuries to the oral cavity and gastrointestinal tract.	2A Major	Yes	3 Occasional The danger has been observed and detected before as described [44]	5 Very severe Fo reign bodies can cause significant injuries to the oral cavity and gastrointestinal tract.	1 Existing detection measures At the end of the process there is a metal detector and an X-ray machine for the detection of any foreign bodies	15	Yes S=5

Stage	Hazard	Control measures	4x4 matrix model				FMEA model				
			Probability	Severity	Risk	SIG	Probability	Severity	Detection	CI	SIG
	(Q) Residual iron salts (ferrous gluconate or lactate) in excessive amounts	Iron salts control (110ppm)	2 Possible to occur in time if not corrected according to the organization's history.	B Severe not imminent [80]	2B Medium	Yes	2 Unlikely These correspond to extremely isolated incidents according to the organization's history.	5 Very severe Failure in the process potentially affects food safety. It involves legal breaches or damage to the consumer's health as described [80]	1 Existing detection measures. Dosage control.	10	Yes<br><br>S=5
	(B) Microorganisms (*Escherichia, Clostridium.*, etc.)	Absence of bad odors and flavors.	2 Possible to occur in time if not corrected [18,24]	A Severe Imminent and immediate danger of death or severe illness as described [40]	2A Major	Yes	3 Occasional The danger has been observed and detected before as described [18, 24]	5 Very severe Imminent and immediate dan-ger of death or severe illness as described [40]	1 Existing detection measures. Absence of bad odors and flavors.	15	Yes<br><br>S=5

Stage	Hazard	Control measures	4x4 matrix model				FMEA model				
			**Probability**	**Severity**	**Risk**	**SIG**	**Probability**	**Severity**	**Detection**	**CI**	**SIG**
III.- Sterilization	(B) Survival of heat-resistant bacteria (sporulated) if the process is inadequate	Temperature: 121ºC Time: >15 minutes	2 Possible to occur in time if not corrected [18,24]	A Severe Imminent and immediate danger of death or severe illness as described [40]	2A Major	Yes	3 Occasional The danger has been observed and detected before as described [18,24]	5 Very severe Imminent and immediate dan-ger of death or severe illness as described [40]	1 Existing detection measures. Control of temperature and time.	15	Yes S=5

P=Physical hazard; C=Chemical hazard; B=Biological hazard; CI= Criticality Index; S=Severity

Table S7. CCP and stricter PRP determination after applying 4x4 matrix and FMEA model in specific stages of Californian-style black olive processing

Stage/Hazard	4x4 Risk matrix	FMEA Risk method*	Q1	Q2	Q3	Q4	CCP/Stricter PRP
I.- Storage in fiberglass tanks with acidified brine and air blowing<br><br>(P) Foreign bodies	Major	CI=15<br>S=5	Yes<br>(PM)	-	-	-	Stricter PRP
I.- Storage in fiberglass tanks with acidified brine and air blowing<br><br>(C) Heavy metals	Medium	CI=15<br>S=5	Yes<br>(WCP)	-	-	-	Stricter PRP
I.- Storage in fiberglass tanks with acidified brine and air blowing<br><br>(B) Microorganisms (*Escherichia, Salmonella, Clostridium…*)	Major	CI=15<br>S=5	No	Yes	Yes<br>(ST)	-	**CCP
II.- Oxidation, lye treatment, and color fixation in aeration tanks<br><br>(P) Foreign bodies	Major	CI=15<br>S=5	Yes<br>(PM)	-	-	-	Stricter PRP
II.- Oxidation, lye treatment, and color fixation in aeration tanks<br><br>(C) Residual iron salts (ferrous gluconate) in excessive amounts	Medium	CI=10<br>S=5	No	Yes	No	Yes	CCP
II.- Oxidation, lye treatment, and color fixation in aeration tanks	Major	CI=15	No	Yes	Yes	-	**CCP

Stage/Hazard	4x4 Risk matrix	FMEA Risk method*	Q1	Q2	Q3	Q4	CCP/Stricter PRP
(B) Microorganisms (*Escherichiai, Salmonella, Clostridium…*)		S=5			(ST)		
III.- Sterilization<br><br>(B) Survival of heat-resistant bacteria (sporulated) if the process is inadequate	Major	CI=15<br><br>S=5	No	Yes	No	Yes	CCP

P=Physical hazard; C=Chemical hazard; B=Biological hazard; ST=Sterilization; WCP= Water control plan; PM=Preventive maintenance.

*Risk: CI=Criticality Index; S=Severity

**Consider whether the control measure at this step works in combination with a control measure at another step to control the same hazard, in which case both steps should be considered as CCPs.
